# Enhanced Synaptic Transmission in the Extended Amygdala and Altered Excitability in an Extended Amygdala to Brainstem Circuit in a Dravet Syndrome Mouse Model

**DOI:** 10.1523/ENEURO.0306-20.2021

**Published:** 2021-06-16

**Authors:** Wen Wei Yan, Maya Xia, Jeremy Chiang, Alyssa Levitt, Nicole Hawkins, Jennifer Kearney, Geoffrey T. Swanson, Dane Chetkovich, William P. Nobis

**Affiliations:** 1 Department of Neurology, Vanderbilt University Medical Center, Nashville, Tennessee 37232; 2 Department of Pharmacology, Northwestern University Feinberg School of Medicine, Chicago, Illinois 60611

**Keywords:** bed nucleus of the stria terminalis, Dravet syndrome, excitability, extended amygdala, parabrachial nucleus

## Abstract

Dravet syndrome (DS) is a developmental and epileptic encephalopathy with an increased incidence of sudden death. Evidence of interictal breathing deficits in DS suggests that alterations in subcortical projections to brainstem nuclei may exist, which might be driving comorbidities in DS. The aim of this study was to determine whether a subcortical structure, the bed nucleus of the stria terminalis (BNST) in the extended amygdala, is activated by seizures, exhibits changes in excitability, and expresses any alterations in neurons projecting to a brainstem nucleus associated with respiration, stress response, and homeostasis. Experiments were conducted using F1 mice generated by breeding 129.Scn1a^+/−^ mice with wild-type C57BL/6J mice. Immunohistochemistry was performed to quantify neuronal c-*fos* activation in DS mice after observed spontaneous seizures. Whole-cell patch-clamp and current-clamp electrophysiology recordings were conducted to evaluate changes in intrinsic and synaptic excitability in the BNST. Spontaneous seizures in DS mice significantly enhanced neuronal c-*fos* expression in the BNST. Further, the BNST had altered AMPA/NMDA postsynaptic receptor composition and showed changes in spontaneous neurotransmission, with greater excitation and decreased inhibition. BNST to parabrachial nucleus (PBN) projection neurons exhibited intrinsic excitability in wild-type mice, while these projection neurons were hypoexcitable in DS mice. The findings suggest that there is altered excitability in neurons of the BNST, including BNST-to-PBN projection neurons, in DS mice. These alterations could potentially be driving comorbid aspects of DS outside of seizures, including respiratory dysfunction and sudden death.

## Visual Abstract

**Figure:**
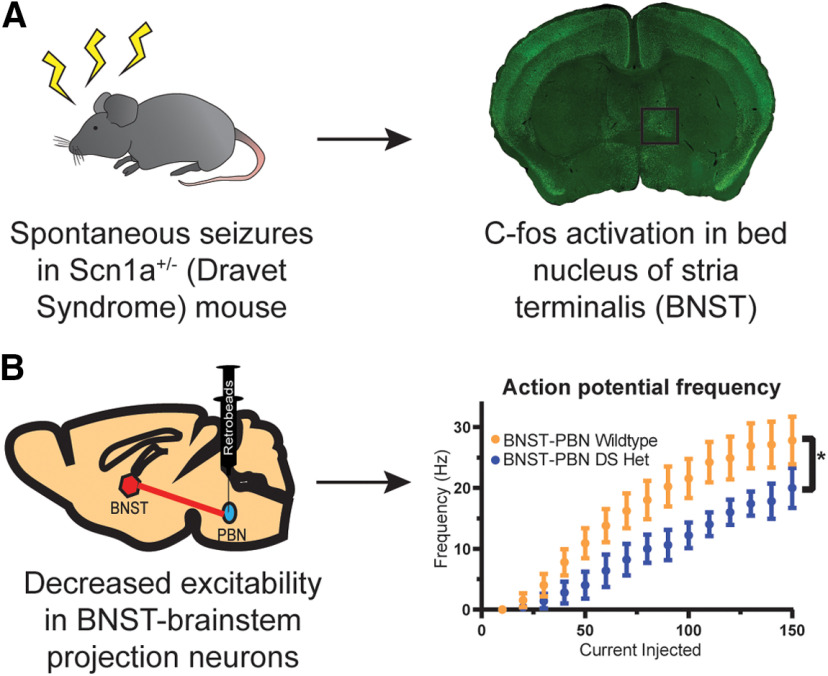

## Significance Statement

Dravet syndrome (DS) is a developmental and epileptic encephalopathy with an increased risk of sudden death. We determined that there are alterations in a subcortical nucleus, the bed nucleus of the stria terminalis (BNST) of the extended amygdala, in a murine DS model. The BNST is involved in stress, anxiety, feeding, and respiratory function. We found enhanced activation in the BNST after seizures and alterations in basal synaptic neurotransmission, with enhanced spontaneous EPSC and decreased spontaneous IPSC events. Evaluating those neurons that project to the parabrachial nucleus, a nucleus with multiple homeostatic roles, we found them to be hypoexcitable in DS. Alterations in BNST to brainstem projections could be implicated in comorbid aspects of DS, including respiratory dysfunction and sudden death.

## Introduction

Dravet syndrome (DS) is a developmental and epileptic encephalopathy with multiple neuropsychiatric comorbidities and an increased risk of sudden death (Gataullina and Dulac, 2017). Most cases arise because of a *de novo* variant in the *scn1a* gene, encoding the sodium channel α subunit Na_V_1.1. Seizures, while usually present early and often being refractory, are not the only manifestation of the disease. Other common comorbidities can be just as impactful on patients and caregivers and include psychomotor delay, cognitive disability, mood disorders, dysautonomia, nutritional issues, and a significant risk of sudden unexpected death in epilepsy (SUDEP; Villas et al., 2017).

Murine models of DS recapitulate many aspects of human disease including severe epilepsy, cognitive and social deficits, and sudden death (Kalume et al., 2013; Lopez-Santiago and Isom, 2019). Work using these models has begun to unravel the neurophysiological alterations in DS that may be underpinnings of these comorbidities. Cardiac abnormalities and peri-ictal breathing issues have been noted in patients and murine models, with more recent work implicating respiratory decline in SUDEP in DS mice. While a major hypothesis proposes that spreading depolarization and subsequent brainstem depression may trigger respiratory decline (Bernard, 2015); evidence of interictal breathing deficits in DS patients and mice suggests that direct alterations in brainstem respiratory centers or their subcortical projecting neurons may exist (Kim et al., 2018). Indeed, recent work shows altered excitability in a region critical for respiratory control and chemosensation (Kuo et al., 2019). It is also possible, yet largely unexplored, that alterations in subcortical projections to these brainstem nuclei may cause deficits as well, with potential far-reaching consequences that could contribute to comorbidities such as temperature dysregulation, mood disorders, feeding, cardiac and respiratory dysfunction, and risk for sudden death. Such alterations may be intrinsic to the subcortical structure or driven by seizures and upstream hyperexcitability.

Despite impressive study of DS mouse models and epilepsy, the neuronal basis for seizures in DS is still debated (Isom, 2014); however, it appears to critically involve forebrain disinhibition (Cheah et al., 2012; Favero et al., 2018; Yuan et al., 2019). Multiple electrophysiological studies demonstrate impaired firing of inhibitory neurons, and selective deletion of Scn1a in inhibitory neurons alone is sufficient to cause seizures and premature death (Cheah et al., 2012). Clearly, intrinsic deficits in cortical inhibition are driving hyperexcitability; however, little work has been performed on the electrophysiological changes in downstream subcortical targets (Derera et al., 2017; Kuo et al., 2019).

A recent study found that targeting a subcortical structure in DS mice, a nucleus of the extended amygdala, could have a significant effect on seizure-induced apneas and mortality because of SUDEP (Bravo et al., 2020). This important study suggests that alterations in a subcortical circuit may be driving an important aspect of DS respiratory dysfunction during seizures. In the present study, we aimed to determine whether another nucleus of the extended amygdala, the bed nucleus of the stria terminalis (BNST), is activated by seizures in DS animals, whether there exists any synaptic or intrinsic excitability changes in this nucleus, and whether neurons projecting to a brainstem structure important for both respiratory function and also overall homeostasis is altered, which could have implications for mechanisms and treatment of important comorbidities in DS.

## Materials and Methods

### Mouse husbandry and genotyping

Animal care and experimental procedures were approved in accordance with the National Institutes of Health *Guide for the Care and Use of Laboratory Animals*. Mice were group housed in a mouse facility under standard laboratory conditions (14/10 h light/dark cycle) and had access to food and water *ad libitum*.

Scn1a^tm1Kea^ mice were obtained (Miller et al., 2014). The line has been maintained as a coisogenic strain by continuous backcrossing of null heterozygotes to 129 (129.Scn1a^+/−^). 129S6/SvEvTac mice (stock #129SVE) were obtained from Taconic. For experiments, F1 mice were produced by breeding heterozygous 129.Scn1a^+/−^ mice with wild-type (WT) C57BL/6J mice (stock #000664, The Jackson Laboratory). Mice were ear tagged and tail biopsied between postnatal day 14 (P14) and P20. At ∼P21, F1 mice were weaned into holding cages containing four to five mice of the same sex and age for experiments.

### Monitoring of spontaneous seizure activity and sudden death

Animals were genotyped and ear tagged from P14 to P20. During this time, mice were also brought to an isolated room in the laboratory for handling each day to acclimate. Mice were kept in their home cage with *ad libitum* access to food and water during the daylight cycle. From P19 to P35, mice were monitored for tonic–clonic convulsive seizures in DS animals. After a DS mouse survived an observed seizure, the DS animal that had the seizure, and a DS littermate and a wild-type littermate, both of which did not have any observed seizures, were killed. Because c-*fos* expression peaks 1–2 h after stimulus onset (Hsieh and Watanabe, 2000), animals were killed 90 min after the experimenter witnessed a seizure, and their ages were recorded.

**Figure 1.**
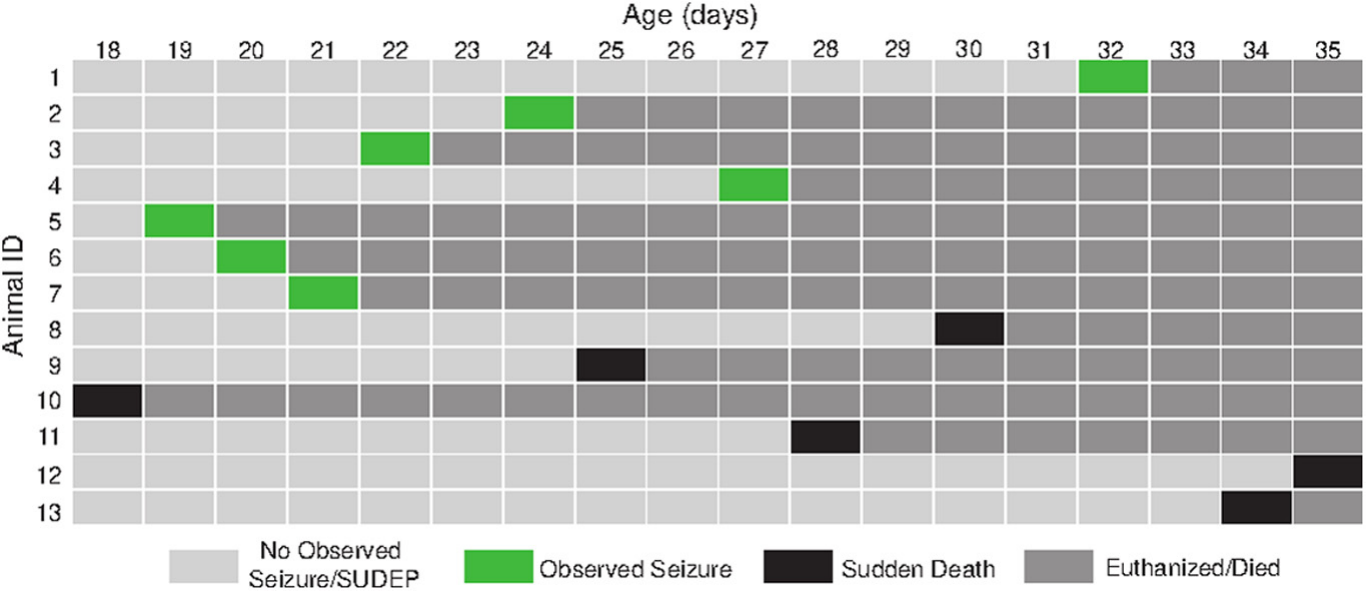
Seizures and sudden deaths captured from 18 to 35 d of age in DS mice. Light gray, No seizures or sudden deaths captured; green, observed spontaneous seizure in home cage, mice killed after capturing a seizure to stain for c-*fos* expression in the BNST; black, sudden death resulting in death of mouse; dark gray, mouse killed or died from SUDEP. Fifteen DS mice were monitored with a sudden death rate of 40%.

Animals were also monitored for sudden death. DS mice that died in their home cages in the mouse facility were marked as having experienced sudden death, and their ages of death were recorded (Fig. 1).

### c-*fos* fluorescent immunohistochemistry

Mice were deeply anesthetized with isoflurane and transcardially perfused with ice-cold PBS followed by 4% paraformaldehyde (PFA). Brains were removed and postfixed for 48 h at 4°C. Coronal sections (30 μm) were made using a vibratome (Leica Biosystems). Antigen retrieval was performed with 10 mm sodium citrate, pH 6.0, for 20 min at 80°C. Slices were cooled, washed with PBS, and immersed in blocking solution (5% normal goat serum, 0.65% w/v BSA, and 0.3% Triton X-100 in PBS) for 1 h at room temperature with gentle agitation. The primary antibody (c-*fos*; Abcam) was diluted in blocking solution and applied overnight at 4°C. Sections were then washed three times for 5 min each and incubated with fluorescently labeled secondary antibodies (Thermo Fisher Scientific) in blocking solution for 1 h at room temperature. Sections were washed an additional three times, with DAPI included in the final wash. Tissues were mounted on glass slides using PermaFluor (Thermo Fisher Scientific). Images were acquired on a microscopy camera (model DFC290, Leica Biosytems). The same acquisition parameters and alterations to brightness and contrast in ImageJ were used across all images within an experiment. Images were thresholded using the striatum image for each genotype as a negative control. Following thresholding, positive cells within the BNST were manually counted using ImageJ by a blinded reviewer. All numbers are reported as a single averaged value for each dorsal BNST (dBNST) and then averaged for each animal.

### Acute slice preparation

Acute brain slices were prepared from P19 to P30 heterozygous and wild-type littermates of either gender, in accordance with Institutional Animal Care and Use Committee-approved protocols. Mice were decapitated under isoflurane, and their brains were removed quickly and placed in an ice-cold sucrose-rich slicing ACSF containing 85 mm NaCl, 2.5 mm KCl, 1.25 mm NaH_2_PO_4_, 25 mm NaHCO_3_, 75 mm sucrose, 25 mm glucose, 10 m dl-APV, 100 μm kynurenate, 0.5 mm Na l-ascorbate, 0.5 mm CaCl_2_, and 4 mm MgCl_2_. Sucrose-ACSF was oxygenated and equilibrated with 95% O_2_/5% CO_2_. Hemisected coronal slices (300 μm) were prepared using a vibratome (model VT1200S, Leica Biosystems). Slices were transferred to a holding chamber containing sucrose-ACSF warmed to 30°C and slowly returned to room temperature over the course of 15–30 min. Slices were then transferred to oxygenated ACSF at room temperature containing 125 mm NaCl, 2.4 mm KCl, 1.2 mm NaH_2_PO_4_, 25 mm NaHCO_3_, 25 mm glucose, 2 mm CaCl_2_, and 1 mm MgCl_2_, and were maintained under these incubation conditions until recording.

### Electrophysiological recordings

Slices were transferred to a submerged recording chamber continuously perfused at 2.0 ml/min with oxygenated ACSF maintained at 30 ± 2°C. Neurons in the dBNST were identified using infrared differential interference contrast on a microscope (Slicescope II, Scientifica). Whole-cell patch-clamp recordings were performed using borosilicate glass micropipettes with tip resistance between 3 and 6 MΩ. Signals were acquired using an amplifier (Axon Multiclamp 700B, Molecular Devices). Data were sampled at 10 kHz and low-pass filtered at 3 kHz. Access resistances ranged between 5 and 24 MΩ and were continuously monitored. Changes >20% from the initial value were excluded from data analyses. Series resistance was uncompensated. Data were recorded and analyzed using pClamp 11 (Molecular Devices).

Current clamp was performed using a potassium gluconate-based intracellular solution containing the following (in mm): 135 K-gluconate, 5 NaCl, 2 MgCl_2_, 10 HEPES, 0.6 EGTA, 4 Na_2_ATP, and 0.4 Na_2_GTP, pH 7.3, at 285–290 mOsm). Input resistance was measured immediately after breaking into the cell and was determined from the peak voltage response to a −5 pA current injection. Following stabilization and measurement of the resting membrane potential, current was injected to hold all cells at a membrane potential of −65 mV, maintaining a common membrane potential to account for intercell variability. Changes in excitability were evaluated by measuring action potential (AP) dynamics and the number of action potentials fired at increasing 10 pA current steps (−150 to 150 pA). The action potential threshold and the amount of current required to fire an action potential (rheobase) were assessed through a ramp protocol of 120 pA/1 s. Parameters related to AP shape, which included AP height, AP duration at half-maximal height (AP half-width), and time to fire an AP (AP latency), were calculated from the first action potential fired during the *I–V* plot. Hyperpolarization sag was calculated as the difference between the initial maximal negative membrane potential and the steady-state current after negative current injection. Neuron types were determined based on the classifications of Hammack et al. (2007) and Rodríguez-Sierra et al. (2013).

For the assessment of spontaneous activity, a cesium methanesulfonate-based intracellular solution (in mm: 135 Cs-methanesulfonate, 10 KCl, 1 MgCl_2_, 0.2 EGTA, 4 MgATP, 0.3 Na_2_GTP, 20 phosphocreatine, and 10 QX-314, pH 7.3, at 285–290 mOsm) was used. Cells were voltage clamped at −65 mV to monitor spontaneous excitatory postsynaptic currents (sEPSCs) and held at +10 mV to isolate spontaneous inhibitory postsynaptic currents (sIPSCs). sIPSCs were measured in 120 s blocks, excluding <5 pA events. Analysis was performed using MiniAnalysis (Synaptosoft).

For evoked experiments, postsynaptic currents were evoked by electrical stimulation using a monopolar glass electrode placed dorsal to the recorded neuron controlled by a SIU91 constant current isolator (Cygnus Tech). An internal recording solution (in mm: 95 CsF, 25 CsCl, 10 Cs-HEPES, 10 Cs-EGTA, 2 NaCl, 2 Mg-ATP, 10 QX-314, 5 TEA-Cl, and 5 4-aminopyridine, pH 7.3, at 285–292 mOSm) was used. To evaluate evoked EPSCs, picrotoxin (25 μm) was added to the ACSF to block both synaptic and extrasynaptic GABA_A_ receptors to assess postsynaptic glutamate transmission. To assess putative changes in presynaptic release probability, paired-pulse ratio (PPR; amplitude of EPSC2/EPSC1) was recorded at a 50 ms interstimulus interval, while the cells were voltage clamped at −65 mV. Similar experiments were conducted to assess short-term plasticity at GABAergic terminals in the presence of kynurenic acid (3 mm) to block ionotropic glutamate receptors. Since evoked IPSCs (eIPSCs) have slower kinetics, a 150 ms interstimulus interval was used and PPR was calculated as the delta amplitude of IPSC2 divided by the amplitude of IPSC1. NMDA/AMPA ratios were calculated based on the peak AMPAR-mediated EPSC amplitude measured at −70 mV, and the NMDAR-mediated EPSC component measured at 40 ms following electrical stimulation at a 40 mV holding potential, respectively. The reversal potential for NMDA receptors is near 0 mV (Misra et al., 2000; Wyeth et al., 2014), and it has been previously determined that at this time point the AMPA component of the ESPC has completely decayed (Fernandes et al., 2009; Hedrick et al., 2017).

### RNAscope *in situ* hybridization

RNAscope detection kits and cDNA probes were purchased from Advanced Cell Diagnostics (ACD) and were used to visualize mRNA species. All probes were generated against Mus musculus-specific transcripts and included parvalbumin (PV; catalog #429131, ACD; channel 1 probe, Mm-Pvalb target region 2–885; GenBank accession #NM_013645.3) and Scn1a (556181, channel 2, Mm-Scn1a target region 1028–4591; GenBank accession #NM_001313997.1).

Wild-type and DS littermates were killed via an overdose of isoflurane, and brains were immediately extracted into optimal cutting temperature media, and brains were frozen with cryospray. Brains were stored at −80°C overnight, then cut on a cryostat (model VT 1000S, Leica) at 16 μm. Sections were adhered to warm Fisher Plus slides (Thermo Fisher Scientific) and were immediately refrozen in dry ice. Slides were stored at −80°C and fixed with ice-cold 4% PFA for 15 min according to ACD protocol. Slides were then placed in an ethanol dilution series (50%, 70%, 100% ×2) for dehydration. Slides were air dried for 5 min, and an ImmEdge Barrier Pen was used to draw a hydrophobic barrier around each section. The ACD four-solution pretreatment was used to incubate sections for 30 min at room temperature in a water bath. Sections were then incubated with RNAscope probes, and the amplification steps were carried out in accordance with the ACD protocol for the Fluorescent Multiplex Kit. Incubation steps included the following: probe mixtures in the kit recommended dilutions for the C1 channel (parvalbumin mRNA) and the C2 channel (Scn1a mRNA) for 2 h at 40°C, wash ×2, AMP-1 FL reagent for 30 min at 40°C, wash ×2, AMP-2 FL reagent for 15 min at 40°C, wash ×2, AMP-3 FL reagent for 30 min at 40°C, wash ×2, AMP-4 FL alt A or B reagent for 15 min at 40°C, wash ×2, DAPI reagent for 1–2 min at room temperature, remove standing liquid, mount, and immediately coverslip with Invitrogen SlowFade Gold (Thermo Fisher Scientific).

Images were acquired on a microscopy camera (model DFC290, Leica Biosytems) with a 20× objective. The same acquisition parameters and alterations to brightness and contrast in ImageJ were used across all images within an experiment. ImageJ software was used to manually count mRNA signals by a blinded reviewer. All numbers are reported as a single averaged value for each hippocampus and dBNST and then were averaged for each animal.

### Stereotaxic surgeries

Mice were anesthetized with isoflurane (initial dose, 3%; maintenance dose, 1.5%) and injected intracranially with red Retrobeads (Lumafluor). A targeted microinjection of the Retrobeads (100 nl) was carried out into the lateral parabrachial nucleus (PBN; coordinates from bregma: medial/lateral, ±1.4 mm, anterior/posterior, −4.9 mm; dorsal/ventral, 3.8 mm; Paxinos and Franklin, 2019). All injections were unilateral. Mice were treated with 5 mg/kg injections of ketoprofen for 48 h following surgery. Electrophysiological recordings were made from these injected animals 48–72 h after injection.

### Experimental design and statistical analysis

The number of animals used and the number of cells evaluated are noted in the Results section for each experiment. Animals of either sex were used during this study, and potential sex-specific differences were evaluated. We did not assume a Gaussian distribution; as such, all comparisons are nonparametric unpaired *t* tests (Mann–Whitney *U* test) used to determine statistical significance. Mean ± SEM values are provided throughout the text. Scatter plots are shown for all data, with mean and SEM graphically depicted. Outliers were removed if values were outside the 95% confidence interval, and it is noted in the text when outliers were removed.

## Results

### Spontaneous seizures induce c-*fos* expression in the dBNST in DS mice

**Figure 2.**
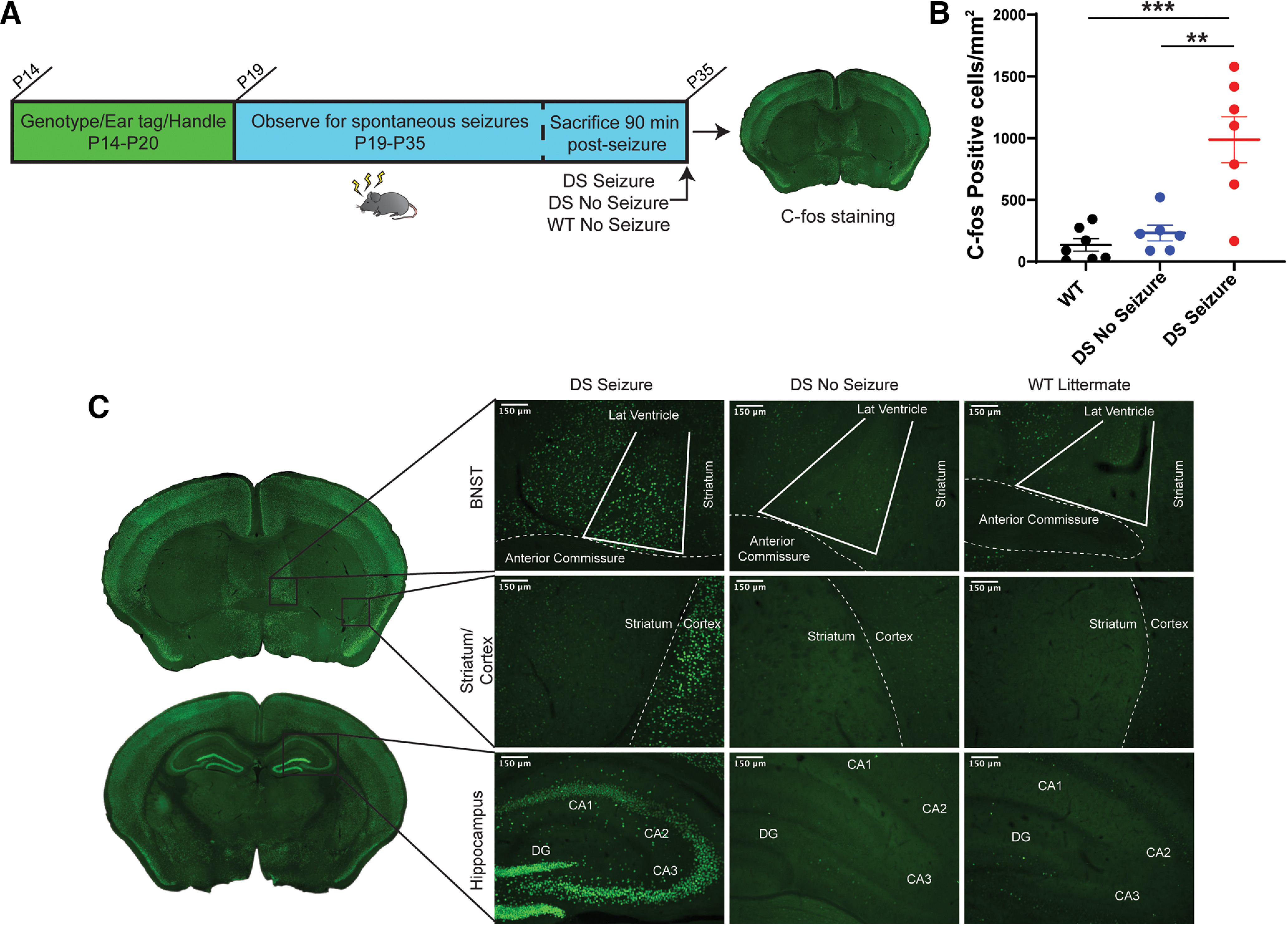
c-*fos* expression in dBNST increases after spontaneous seizures in DS mice. ***A***, Schematic showing timeline of home-cage monitoring for seizures and immunohistochemistry for c-*fos* in dBNST of DS and WT littermates. ***B***, Scatter plot showing a significant increase in the mean number of c-*fos*-activated cells in dBNST sections of DS mice after a spontaneous seizure (DS seizure, *n* = 7; DS no seizure, *n* = 6; WT, *n* = 7; DS seizure vs WT, *p* = 0.0004; DS seizure vs DS no seizure, *p* = 0.0021; DS no seizure vs WT, *p* = 0.1263). ***C***, Coronal sections denote dBNST, striatum, and hippocampus regions. Representative images of c-*fos* in DS mice 1.5 h postseizure, DS littermates with no seizure, and WT littermates with no seizure. Striatum served as a negative control and hippocampus as a positive control for c-*fos* expression. ***p < 0.001, **p < 0.01.

We first wanted to determine whether the BNST was activated by seizures in DS mice. To do this, we analyzed expression patterns of the immediate early gene c-*fos* following seizures in the dBNST. Previous studies consistently show robust c-*fos* expression in hippocampal regions following seizures; however, nuclei of the extended amygdala have not yet been evaluated (Dutton et al., 2017; Huffman et al., 2018). We monitored naive Scn1a^+/−^ mice for spontaneous seizures in their home cage with wild-type littermates from P19 to P35 (Fig. 2*A*). None of the wild-type littermates experienced a seizure. Following a spontaneous seizure, there was a significant increase in c-*fos*-positive cells in the dBNST compared with wild-type littermates and DS littermates that did not have an observed seizure (DS seizure: 987 ± 186.1 cells/mm^2^, *n* = 7; DS no seizure: 231.6 ± 64.59 cells/mm^2^, *n* = 6; WT: 134.4 ± 50.14 cells/mm^2^, *n* = 7; DS seizure vs WT: *p* = 0.0004; DS seizure vs DS no seizure: *p* = 0.0021; Fig. 2*B*,*C*). There was no significant difference in c-*fos* activation at baseline levels in DS mice and WT mice (DS no seizure vs WT: *p* = 0.1263; Fig. 2*B*,*C*). In contrast, there was no significant increase in c-*fos* cells in the dorsal striatum in these animals, a region like the BNST that has stress responsiveness (Perrotti et al., 2004). This increased activation appears to result from seizures, as there was no difference in dBNST c-*fos* activation at baseline with just stress of handling (Fig. 2*B*,*C*).

### Altered postsynaptic receptor composition in the dBNST in DS mice without changes in release probability

**Figure 3.**
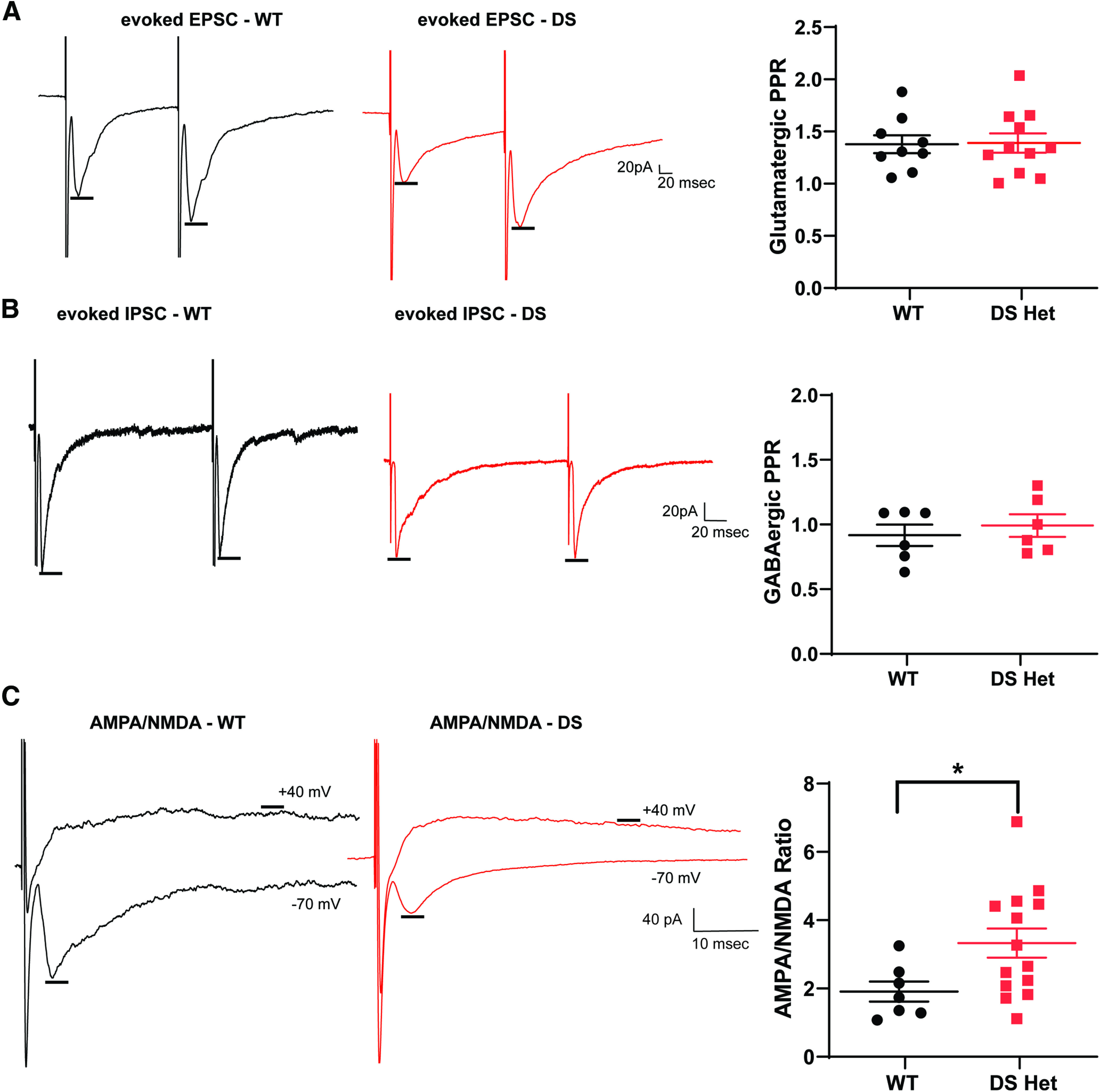
Postsynaptic receptor composition in dBNST is altered in DS mice, but there are no changes in release probability. ***A***, Representative evoked EPSC trace of WT (black) and DS (red) neurons in a bath with GABA_A_ receptor blockers. Scatter plot showing no significant change in excitatory paired-pulse ratios (WT, *n* = 9; DS, *n* = 11; *p* > 0.99). ***B***, Representative eIPSC trace of WT and DS neurons in a bath with ionotropic glutamate receptor blockers. Scatter plot showing no change in inhibitory paired pulse ratios (WT, *n* = 6; DS, *n* = 6; *p* = 0.59). ***C***, Representative traces of AMPA receptor- and NMDA receptor-mediated EPSCs in WT and DS neurons. Scatter plot showing a significant increase in AMPA/NMDA ratio in DS mice (WT, *n* = 7; DS, *n* = 14; *p* = 0.04). *p < 0.05.

As an initial electrophysiological investigation into potential alterations in dBNST neurons in DS mice, we measured evoked synaptic properties (Fig. 3*A*,*B*). First, we evaluated synaptic facilitation, a form of short-term plasticity. We bath applied either GABA_A_ or ionotropic glutamate receptor blockers to isolate excitatory or inhibitory evoked events. We evoked pairs of postsynaptic currents by delivering electrical stimulation, allowing us to measure a PPR. PPR alterations reflect changes in the probability of presynaptic neurotransmitter release (Regehr, 2012). Such alterations are seen in the dentate gyrus in other DS mouse models (Tsai et al., 2015). We found no changes in excitatory PPR (WT: 1.38 ± 0.09, *n* = 9 cells from 6 mice, DS: 1.39 ± 0.9, *n* = 11 cells from 7 mice; *p* > 0.99; Fig. 3*A*) or inhibitory PPR (WT: 0.92 ± 0.08, *n* = 6 cells from 5 mice; DS: 0.99 ± 0.09, *n* = 6 cells from 5 mice; *p* = 0.59; Fig. 3*B*) in the dBNST. We further investigated the contribution of postsynaptic receptors to these evoked excitatory currents. We measured AMPA receptor-mediated EPSCs by voltage clamping the cell at −70 mV, and NMDA receptor-mediated currents by holding the same cell at +40 mV. This allowed us to calculate the AMPA/NMDA ratio for each cell. Changes in AMPA/NMDA ratio are a potential hallmark for evidence of plasticity and are seen after learning and memory tasks, but also in pathologic situations such as temporal lobe epilepsy models (Amakhin et al., 2017). We found a significant increase in this ratio in DS mice in dBNST neurons (WT: 1.9 ± 0.3, *n* = 7 cells from 5 mice; DS: 3.3 ± 0.4, *n* = 14 cells from 8 mice; *p* = 0.04; Fig. 3*C*).

### Decreased spontaneous inhibitory and enhanced excitatory neurotransmission in DS mice

**Figure 4.**
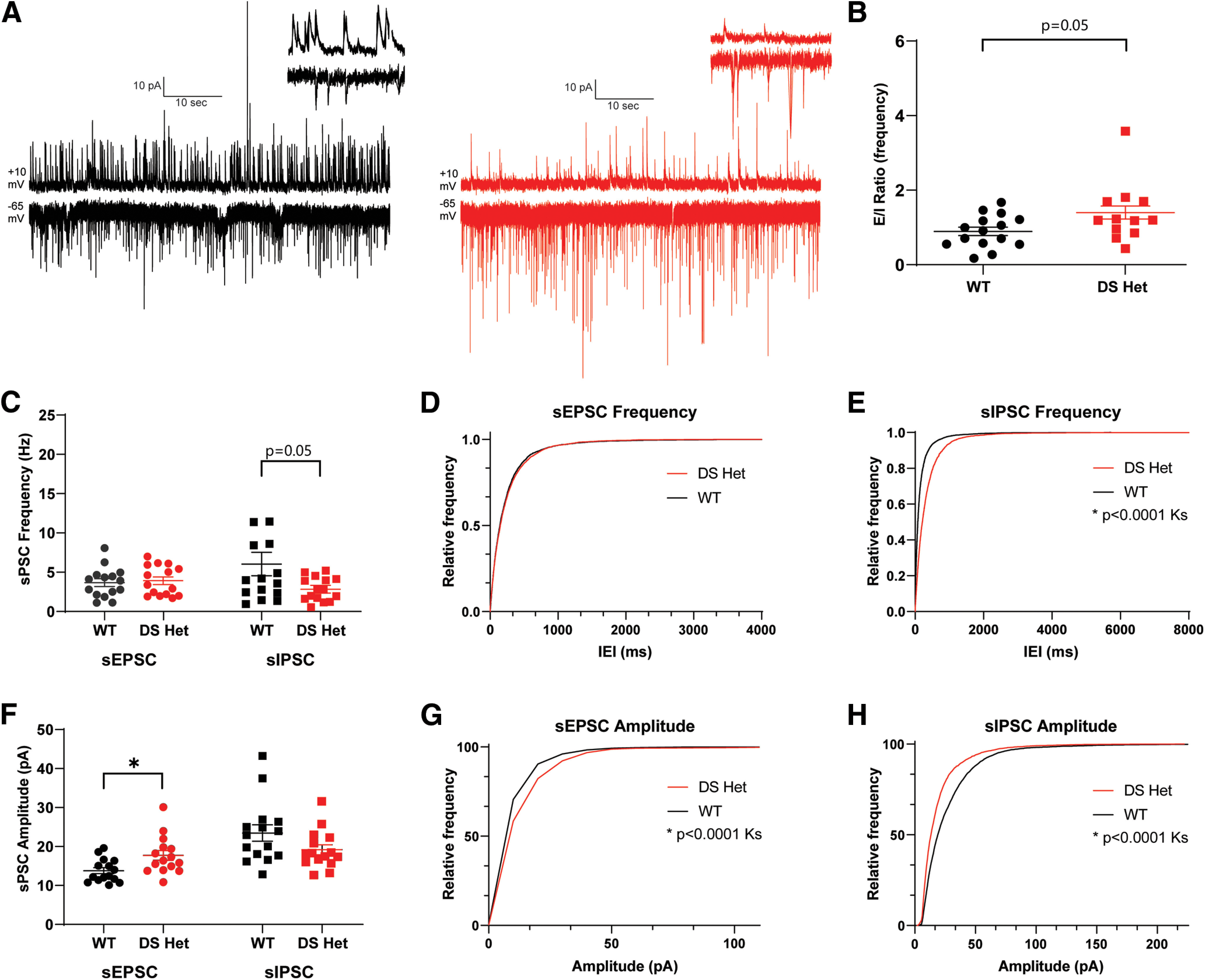
Spontaneous inhibitory neurotransmission is decreased in DS mice, while spontaneous excitatory neurotransmission is enhanced. ***A***, Example 60 s traces of WT (black) and DS (red) recordings of sIPSCs at +10 mV (top) and of sEPSCs at −65 mV (bottom). Inset, 2 s trace (top right). ***B***, Scatter plots showing significant increase in E/I ratio of the frequency of sEPSCs to sIPSCs in DS mice (*p* = 0.056). ***C***, Scatter plots showing no significant change in sEPSC frequency (WT, *n* = 15; DS, *n* = 15; *p* = 0.68) and a significant decrease in sIPSC frequency in DS mice (*p* = 0.046). ***D***, ***E***, Cumulative probability plots for sEPSC frequency showing no change, with sIPSC frequency showing a significant decrease in DS mice. ***F***, Scatter plots showing a significant increase in sEPSC amplitude (*p* = 0.016) and a decrease in sIPSC amplitude in DS mice (*p* = 0.059). ***G***, ***H***, Cumulative probability plots for sEPSC amplitude showing a significant increase, with sIPSC amplitude showing a significant decrease in DS. *p < 0.05.

Excitatory and inhibitory (E-I) balance is altered in DS mice in cortical and hippocampal regions; to examine the effect of this on the dBNST, we measured both sEPSCs and sIPSCs. Using a Cs-methanesulfonate internal solution (see Materials and Methods), we could measure both sEPSCs when voltage clamped at −65 mV and spontaneous sIPSCs in the same cells when voltage clamped at +10 mV (Fig. 4*A*). While there were no changes in the frequency of excitatory events (WT: 3.7 ± 1.9 Hz, *n* = 15 cells from 7 mice; DS: 3.9 ± 0.5 Hz, *n* = 15 cells from 8 mice; *p* = 0.68; Fig. 4*C*; all experiments in this section are from the same number of mice/cells), there was a significant increase in amplitude of spontaneous events (WT, 13.8 ± 0.77 pA; DS, 17.7 ± 1.25 pA; *p* = 0.016; Fig. 4*F*,*G*). This is consistent with the increase in AMPA/NMDA, which could result from either a decrease in NMDA synaptic currents or a selective increase in AMPA synaptic currents. Spontaneous inhibitory events in the dBNST were diminished, with a trend for a decrease in sIPSC frequency (WT: 4.8 ± 0.9 Hz, *n* = 14 cells from 7 mice with one high outlier removed; DS: 2.8 ± 0.4 Hz; *p* = 0.056; Fig. 4*C*,*E*).

Given the heterogeneous nature of BNST inputs and intrinsic circuits, a decrease in the number of release sites could be driving this change (Li et al., 2012). There was also a trend for a decrease in the sIPSC amplitude in DS mice (WT, 23.5 ± 2.1 pA; DS, 19.2 ± 1.3 pA; *p* = 0.09; Fig. 4*F*,*H*). Using this approach to look at both sEPSC and sIPSC within neurons, we calculated an E-I balance for each, determining that there was a trend for an increase in DS mice in the frequency of excitatory to inhibitory PSCs (WT, 0.89 ± 0.11; DS, 1.38 ± 0.80; *n* = 13 from 7 mice with two high outliers removed; *p* = 0.059; Fig. 4*B*). Overall, there is an increase in basal synaptic drive in the dBNST in DS mice.

### Minor changes in overall intrinsic excitability of dBNST neurons in DS mice

**Figure 5.**
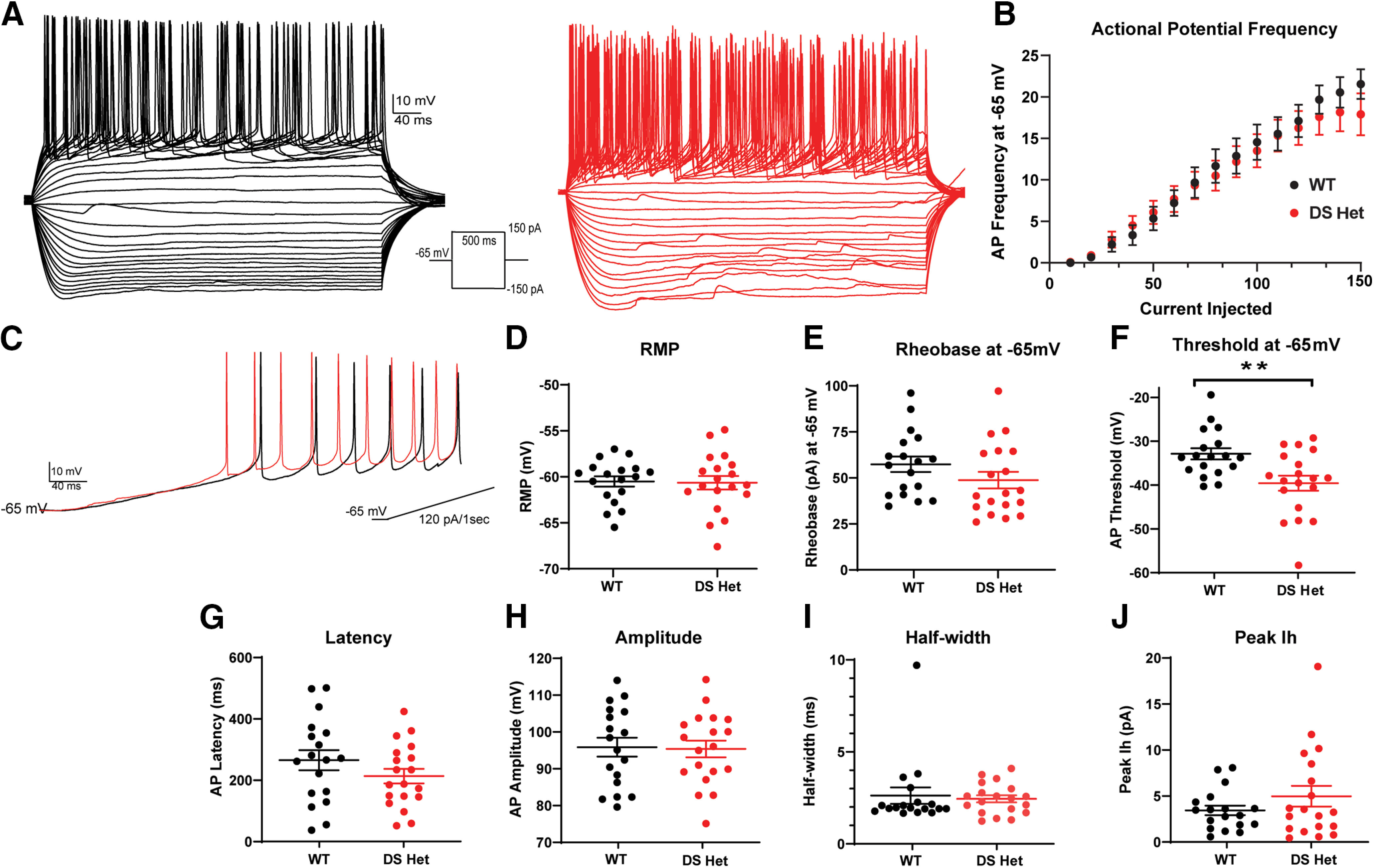
The overall intrinsic excitability of neurons is slightly enhanced in the dBNST of DS mice as a result of lower action potential threshold. ***A***, Representative traces of action potentials during current injection protocol in WT (black) and DS (red) mice. ***B***, Summary plot showing no difference in action potential frequency after current injection. ***C***, Representative trace of ramp current injection in WT (black) and DS (red) mice. ***D***, ***E***, Scatter plots showing no difference in resting membrane potential (WT, *n* = 18; DS, *n* = 19; *p* = 0.85) and rheobase (*p* = 0.12). ***F***, Scatter plot showing significantly lower action potential threshold in DS mice (*p* = 0.007). ***G*–*J***, Scatter plots showing no changes in action potential latency, amplitude, half-width, and peak *I*_h_ in WT and DS mice. Scn1a expression in the BNST is unlikely to play a role in excitability changes, as we find low levels of both Scn1a and parvalbumin in WT and DS mice (Extended Data Figure 5-1). **p < 0.01.

The Na_V_1.1 channel is a component of many inhibitory neurons, and its loss of function in DS models results in diminished excitability and altered AP dynamics. To investigate whether alterations exist in intrinsic excitability of the largely GABAergic population of both projection and interneurons in the dBNST, we first used brief voltage steps to measure intrinsic properties including input resistance (WT: 625 ± 39.3 MΩ, *n* = 18 cells from 9 mice; DS: 666 ± 75.3 MΩ, *n* = 19 cells from 8 mice; *p* = 0.96; all experiments in this section are from the same number of mice/cells) and capacitance (WT, 49 ± 4.0 pF; DS, 56 ± 7.0 pF; *p* = 0.62) before assessing AP kinetics and excitability in current clamp. After recording the resting membrane potential (RMP; WT, −60.5 ± 0.56 mV; DS, −60.7 ± 0.73 mV; *p* = 0.85; Fig. 5*D*), all measurements were recorded in neurons clamped at −65 mV to account for the slight variabilities of RMPs between neurons. Overall, there was no change in the number of generated APs across the range of current injections (Fig. 5*B*), nor were there changes in the rheobase (minimum current required to elicit AP: WT, 57 ± 4.2 pA; DS, 49 ± 4.5 pA; *p* = 0.12; Fig. 5*E*) between the groups. Similarly, AP latency, amplitude, and half-width were unchanged between groups (Fig. 5*H–J*). Notably, the threshold to fire the first AP was decreased in DS mice (WT, −32.9 ± 1.28 mV; DS, −39.6 ± 1.72 mV; *p* = 0.007; Fig. 5*F*), indicating the slight increase in excitability. The BNST is a heterogeneous population; however, the neurons can be divided based on their intrinsic membrane currents (Egli and Winder, 2003; Hammack et al., 2007). When subdividing into these categories (type I, type II, type III, and other), there were no differences in intrinsic excitability between the WT and DS groups, aside from type I neurons displaying a lower threshold (WT: −32.6 ± 1.56 mV, *n* = 8 cells; DS: −38.1 ± 1.22 mV, *n* = 5 cells; *p* = 0.02). To determine whether excitability changes were influenced by loss of Na_V_1.1 channels in BNST neurons, we evaluated mRNA expression of Scn1a in the BNST using fluorescence *in situ* hybridization (RNAscope), finding low levels of Scn1a and parvalbumin in both WT and DS mice (Extended Data Fig. 5-1).

**Figure 6.**
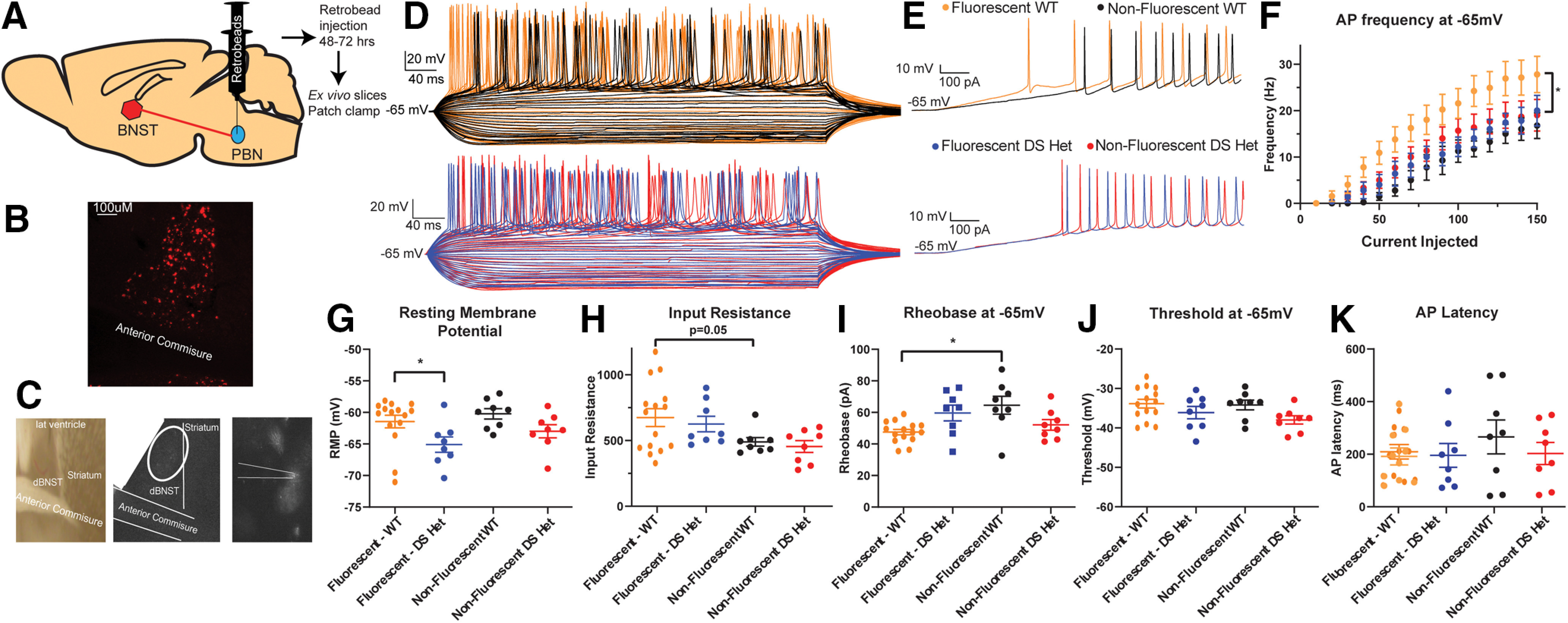
Neurons projecting from the dBNST to PBN are more excitable in WT mice and exhibit hypoexcitability in DS mice. ***A***, Schematic showing unilateral Retrobead injection into PBN. ***B***, Representative image of fluorescent neurons in dBNST following Retrobead injection. ***C***, Representative image of fluorescent neurons being identified for patch clamp. ***D***, Representative traces of action potentials during current injection protocol in WT fluorescent neurons (top, orange), WT nonfluorescent (black), DS fluorescent (bottom, blue), and DS nonfluorescent (red). ***E***, Representative trace of ramp current injection in WT (top) and DS (bottom) mice. ***F***, Summary plot showing dBNST-to-PBN projection neurons in WT mice firing more action potentials and no change in DS neurons across a range of current injections (fluorescent WT, *n* = 15; nonfluorescent WT, *n* = 8; fluorescent DS, *n* = 8; nonfluorescent DS, *n* = 8; *p* < 0.0001, two-way ANOVA). ***G***, Scatter plot showing significant decrease in resting membrane potential of dBNST-to-PBN projection neurons in DS mice compared with WT mice (*p* = 0.013). ***H***, Scatter plot showing a trend for increase in membrane resistance in PBN projecting neurons compared with other neurons in WT mice (*p* = 0.058). ***I***, Scatter plot showing a significant decrease in rheobase in fluorescent neurons compared with nonfluorescent neurons in WT mice (*p* = 0.009). ***J***, ***K***, Scatter plots showing no significant changes in threshold and AP latency. *p < 0.05.

10.1523/ENEURO.0306-20.2021.f5-1 Figure 5-1 RNAScope of Scn1a and PV interneurons in hippocampus and BNST in DS and WT littermates. ***A***, ***C***, Scn1a colocalized with PV expression in the CA1 region of the hippocampus of both DS and WT mice with significantly lower Scn1a expression in DS mice. ***B***, ***D***, Scn1a and PV expression in the BNST is very sparse in both WT and DS mice. ***E***, Quantification of Scn1a and PV expression in the hippocampus. Significant reduction in PV and Scn1a colocalized cells in CA1 hippocampal region of DS mice (WT, *n* = 8; DS, *n* = 4; *p* = 0.0069). Download Figure 5-1, TIF file.

### dBNST neurons projecting to the PBN are a more excitable population than non-PBN-projecting neurons

To determine how changes in the BNST may be influencing downstream targets driving DS comorbidities, we chose to focus on neurons that project to the PBN. The PBN is a brainstem structure involved in arousal, respiratory function, and overall homeostatic functions (Campos et al., 2018). To target these neurons, we stereotaxically injected red Retrobeads unilaterally into the PBN and performed whole-cell current-clamp experiments from both fluorescent and nonfluorescent neurons (Fig. 6*A–C*) 48–72 h after injection. These neurons on average had a higher input resistance (fluorescent: 662 ± 71 MΩ, *n* = 15 cells from 11 mice; nonfluorescent: 490 ± 33 MΩ, *n* = 8 cells from 8 mice; unpaired *t* test, *p* = 0.058; Fig. 6*H*; all experiments in this section are from the same number of mice/cells) and a trend for a larger capacitance (fluorescent, 53 ± 5.8 pF; nonfluorescent, 34 ± 6.8 pF; unpaired *t* test, *p* = 0.1), consistent with BNST projection neurons (Luster et al., 2019). Furthermore, we found that dBNST neurons projecting to the PBN are more excitable—firing more APs across ranges of current injections (two-way ANOVA: *F*_(15,390)_ = 25.83; *p* < 0.0001 for main effect of group; Fig. 6*F*) and with a significantly lower rheobase (fluorescent, 47 ± 1.7 pA; nonfluorescent, 65 ± 5.7 pA; unpaired *t* test, *p* = 0.0009; Fig. 6*H*). Otherwise, there were no differences in RMP, AP kinetics, peak *I*_h_, or threshold in these neurons in wild-type littermates. Previous reports indicate that neurons in the rat and mouse BNST can be categorized into at least three distinct neuronal subtypes based on voltage responses to transient current steps (Egli and Winder, 2003; Hammack et al., 2007). All of these physiologically distinct types of neurons were represented in the population of BNST neurons projecting to the PBN, suggesting that changes in excitability in these projection neurons are not simply an enrichment for one type. The plurality of these were type I neurons (*n* = 6 of 15 cells), but type II neurons (*n* = 2 of 15 cells), type III neurons (*n* = 4 of 15 cells), and other neurons (*n* = 3 of 15) are present. The fact that there is not a strict predominance of a single physiological type in those neurons projecting to the PBN is not unexpected as neuronal morphology, expression of neuropeptide markers, and projection location have not been shown previously to associate with a particular physiological subtype (Rodríguez-Sierra et al., 2013; Silberman et al., 2013; Ch’ng et al., 2019).

### dBNST neurons projecting to the PBN are hypoexcitable in DS mice

To determine how these potentially important projection neurons were altered in DS mice, we similarly performed recordings from dBNST neurons in DS mice unilaterally injected with red Retrobeads in the PBN. Fluorescent neurons, representing those dBNST neurons projecting to the PBN, had a significantly hyperpolarized RMP in DS mice compared with fluorescent neurons in WT mice (fluorescent WT: −61 ± 1.0 mV, *n* = 15 cells from 11 mice; nonfluorescent WT: −60 ± 0.82 mV, *n* = 8 cells from 8 mice; fluorescent DS: −65 ± 1.2 mV, *n* = 8 cells from 6 mice; nonfluorescent DS: −63 ± 1.0, *n* = 8 cells from 6 mice; *p* = 0.05, Tukey’s *post hoc* test; Fig. 6*G*; all experiments in this section are from the same number of mice/cells). Strikingly, dBNST-to-PBN projection neurons in DS mice no longer had an increase in AP fired with respect to current injections, suggesting that these brainstem-projecting neurons have decreased excitability in DS mice (two-way ANOVA and Tukey’s *post hoc* test; fluorescent DS vs nonfluorescent WT, *p* = 0.21; fluorescent DS vs nonfluorescent DS, *p* = 0.74 for main effect of group; Fig. 6).

Other parameters, including AP amplitude, half-width, latency, and peak *I*_h_ were unchanged (Fig. 6*I–K*). In DS mice, dBNST projection neurons are therefore hypoexcitable (hyperpolarized, decreased AP firing) with normal AP kinetics.

## Discussion

The current study was motivated by the relative paucity of investigation of subcortical structures in DS despite the impact that pathology in these regions may have on multiple aspects of DS, including sudden death. We used the Scn1a^+/−^ Dravet model to perform a detailed electrophysiological examination of the dorsal BNST of the extended amygdala, as it receives inputs from cortical and hippocampal regions (Reichard et al., 2017) and has dense outputs to brainstem structures (Dong and Swanson, 2006). This report shows that the dBNST is activated by seizures in DS mice and that there is marked excitatory-inhibitory synaptic imbalance in this region as well as alterations in postsynaptic receptor composition. We further investigated those BNST neurons projecting to the lateral PBN region in the pons and found that these neurons are a physiologically distinct population that are hypoexcitable in DS mice. These results provide evidence for distinct neuroadaptations in the BNST in DS mice and are complementary to the growing literature investigating the importance of subcortical structures in the Dravet model (Kuo et al., 2019; Bravo et al., 2020).

### Enhanced synaptic drive and synaptic strength in the BNST in DS mice

We provide evidence of enhanced synaptic drive in the dorsal BNST in DS mice, with an increase in the amplitude of spontaneous excitatory events and a decrease in both the amplitude and frequency of inhibitory events. We additionally show evidence of postsynaptic changes, with an increase in AMPA/NMDA ratio, which suggests that alterations in postsynaptic glutamate receptors may be driving the increase in amplitude of sEPSCs. This may be driven by an enhanced excitatory drive from cortical pyramidal neurons and the ventral hippocampus, which have increased firing in DS mouse models (Yu et al., 2006; Mistry et al., 2014; Tai et al., 2014), and both project to the BNST. A decrease in inhibitory drive, both the amplitude and frequency of events, could be a counterbalancing effect for the enhanced excitatory drive. Alternatively, neuronal firing of inhibitory inputs could also be diminished. The BNST receives the majority of its inhibitory input from the central amygdala (CeA; Cassell et al., 1999), and, unlike the BNST, the CeA has a large population of PV neurons (Wang et al., 2016), which likely have impaired firing in DS mice (Yu et al., 2006; Tai et al., 2014). Finally, our data strongly suggest that the BNST is highly activated following seizures in DS mice. The frequency of seizures and subsequent strong neuronal activation of this region might be driving alterations in AMPA/NMDA ratio and producing compensatory changes in inhibitory synaptic drive.

### Subtle increase in intrinsic excitability in BNST neurons in DS mice

We found subtle changes in intrinsic excitability overall, with an increase in the intrinsic excitability of dBNST neurons associated with a lowering of threshold to fire an AP. This was, however, not associated with any increase in spike frequency with current injections or other changes in excitability or AP kinetics. The somewhat contradictory nature of this is likely because of the heterogeneity of the BNST neurons and the inability to distinguish between interneurons or projection neurons when doing blind-patching experiments—there may be a population within the dBNST that has a more striking enhancement in intrinsic excitability if they were to be targeted directly. Many of the dBNST neurons are GABAergic, with both interneurons and inhibitory projection neurons. In other brain regions in DS models, inhibitory neurons, particularly PV interneurons, have greatly diminished excitability and changes to AP kinetics. This was not seen overall in the BNST, likely because of the very small to nonexistent population of PV interneurons in this nucleus, as previously reported (Nguyen et al., 2016) and confirmed (Extended Data Fig. 5-1), which was unlikely to be sufficiently sampled by blind patching.

### Decreased excitability in BNST to PBN projection neurons

We were interested in how any changes in the BNST excitability could alter autonomic functioning, breathing, and homeostatic behaviors that are perturbed in DS, so we evaluated dBNST neurons that project to the lateral PBN by using a retrograde tracing approach. We found that these projection neurons are a more excitable population compared with the dBNST as a whole, with a higher input resistance, a lower rheobase, a lower latency to fire APs, and a higher frequency of firing APs with each current injection. These attributes are no longer present in PBN-projecting neurons in DS mice, where these neurons also show a significant hyperpolarizing change in resting membrane potential, implying potential changes in potassium channel currents. These changes may be compensatory and driven by the aforementioned E-I balance increase, or by the epilepsy state itself. Potentially, GABAergic projection neurons to the PBN may have reduced excitability because of the loss of Na_V_1.1 channels (Kalume et al., 2007); however, this seems less likely given the low basal level of Scn1a expression (Extended Data Fig. 5-1). These projection neurons may be preferentially bombarded by repeated cortical seizures, producing changes in intrinsic excitability to counteract this effect and overall diminishing output to the PBN.

### Functional implications

The BNST is a limbic structure implicated in mediating behavioral responses to anxiety and stress, and is intimately interconnected to the larger amygdala circuit, hippocampus, and cortical regions (Lebow and Chen, 2016; Knight and Depue, 2019). The BNST may function as an integrator of these higher-order inputs, affecting its function over the autonomic nervous system, breathing, stress response, and homeostasis through dense projections to hypothalamic, midbrain, and brainstem nuclei, including the periventricular nucleus, ventral tegmental area, periaqueductal gray, nucleus tractus solitarius, PBN, and serotonergic neurons in the dorsal and midbrain raphe (Cassell et al., 1999; Dong and Swanson, 2004, 2006; Pollak Dorocic et al., 2014). In particular, there is evidence that the extended amygdala region may be critically involved in the respiratory dysfunction seen during seizures in DS (Kim et al., 2018; Kuo et al., 2019). Electrical stimulation of the human amygdala reliably produces apneas (Dlouhy et al., 2015; Lacuey et al., 2019; Nobis et al., 2018, 2019). This is likely related to the stimulation of structures of the extended amygdala rather than the lateral regions (Nobis et al., 2018; Rhone et al., 2020). Similarly, seizure spread to these regions may be driving ictal apneas (Dlouhy et al., 2015; Nobis et al., 2019). The involvement of the extended amygdalar regions (CeA and BNST) in ictal apneas may explain why the amygdala at large does not always produce apneas when activated by seizures (Park et al., 2020). The PBN has a known role in the modulation of breathing (Dutschmann and Herbert, 2006; Navarrete-Opazo et al., 2020) and has been targeted to rescue respiratory dysfunction in developmental disorders (Abdala et al., 2016). The BNST input to the PBN can influence respiration (Kim et al., 2013), so it is possible that the decreased intrinsic excitability of BNST projection neurons may produce compensatory changes in the PBN that allow strong stimuli in the form of a seizure to now produce an aberrant response, resulting in apneas. This could work in concert with dysfunction in other brainstem respiratory regions such as the retrotrapezoid nucleus (Kuo et al., 2019), which is more involved in setting respiratory rhythm (Guyenet et al., 2019), with disastrous outcomes leading to ictal breathing suppression and SUDEP. The known role of the lateral PBN in cardiovascular function may also be playing a role (Davern, 2014).

Outside of respiratory and cardiovascular function, the BNST inputs to the PBN have been implicated in anxiety, morphine withdrawal, feeding, and aversion (Mazzone et al., 2018; Luster et al., 2019; Wang et al., 2019). The PBN itself has important roles in other homeostatic functions including arousal and temperature regulation (Campos et al., 2018). Altered extended amygdalar inputs to this critical brainstem region could be driving some other persistent aspects of DS outside of seizures. This highlights the general need to explore subcortical structures in epilepsy models, in part to shed light on the comorbidities of epilepsy states. The few studies that have been performed evaluating these regions in human epilepsy patients show widespread alterations in subcortical network activity, including the brainstem, that correlate with impairments in vigilance and arousal (Englot et al., 2020). This current study emphasizes the need to evaluate these structures in DS and other epilepsy models, particularly in linking circuit dysfunction to other behavioral and physiologic comorbidities seen in epilepsy.

In sum, our results show that the BNST of the extended amygdala is activated in Scn1a^+/−^ mice, and that there are neuroadaptations in this region in DS mice. These adaptations include decreased intrinsic excitability of those neurons projecting to the PBN in the brainstem. These alterations could potentially be driving comorbid aspects of DS outside of seizures, including respiratory dysfunction and sudden death.

## Synthesis

Reviewing Editor: Katalin Toth, University of Ottawa Decisions are customarily a result of the Reviewing Editor and the peer reviewers coming together and discussing their recommendations until a consensus is reached. When revisions are invited, a fact-based synthesis statement explaining their decision and outlining what is needed to prepare a revision will be listed below. The following reviewer(s) agreed to reveal their identity: NONE.

The authors have addressed all the issues raised by the reviewer. There few minor issue to consider:

Lines 373-377. As these lines make the major point of this section. they should be set off as a new sentence, Figure 5F should be called out here, and this key result should be stated in a more positive and definite way than “no longer had any change between non-fluorescent neurons from WT or DS mice”.

## Author Response

Response to reviewers.

Synthesis Statement for Author (Required):

This is a potentially interesting study investigating a mechanism that could contribute to the better

understanding of SUDEP, a poorly understood phenomenon. Both reviewers raised several critical issues

that need to be addressed by the authors. The study needs better definition of the animal model and the

authors need to better differentiate between the immediate effects of the hyperthermia treatment and

spontaneous seizures. The time window they used (24-72 hr) is very close to the induction period that

makes this distinction quite difficult. Febrile seizure paradigm usually use a significantly longer period to

allow the development of recurring (‘spontaneous’) seizures. Seizures occurring in DS het and WT

animals will need to be carefully quantified both during the hyperthermia treatment and the spontaneous

phase.

We appreciate the interest in the work and are thankful for the opportunity to submit a revision to the initial

manuscript. We also thank the editor and reviewers for allowing additional time to perform these

experiments and revisions as covid-related delays and adjustments made it difficult to re-establish our

mouse colony.

We agree that the hyperthermia treatment we employed made the c-fos data interpretation difficult - to this

end, we have added data from animals after a spontaneous seizure without any preceding exposure to a

hyperthermia paradigm (see Figure 1).

We also agree that while the DS model we are using has been widely used by other groups (see Bahceci,

D. et al. (2020), Epilepsy & behavior, 103, p. 106842; Favero, M. et al. (2018), The Journal of

neuroscience, 38(36), pp. 7912-7927; Han, S. et al., Nature, 489(7416), pp. 385-390; Hawkins, N. A. et

al. (2019), Experimental neurology, 311, pp. 247-256; Yuan, Y. et al. (2019), Scientific reports, 9(1), p.

6210), there is certainly potential for variability between laboratories. The data from capturing

spontaneous seizures addresses the concerns that the model is behaving as noted by prior groups. We

additionally present data on rates of sudden death in our animals, which is comparable to what has been

published by other groups (Table 1) In addition, many changes have been made as detailed below to

address the helpful critiques, which we feel makes this a much stronger manuscript.

REV.#1.

1. Hyperthermia treatment. As defined by the Mayo Clinic online: “Heatstroke is a condition caused by

your body overheating, usually as a result of prolonged exposure to or physical exertion in high

temperatures. This most serious form of heat injury can occur if your body temperature rises to 104 F (40

C) or higher. A core body temperature of 104 F (40 C) or higher, obtained with a rectal thermometer, is

the main sign of heatstroke. Altered mental state or behavior, confusion, agitation, slurred speech,

irritability, delirium, seizures, and coma can all result from heatstroke.” Based on this standard definition,

treatment of mice with a core body temperature of 42.5 degrees C causes heat stroke, which in turn can

cause seizures and other neuropathologies. Therefore, the hyperthermia stimulus used here is actually a

heat stroke treatment. The spontaneous seizures that follow this heat stroke treatment in 24 to 72 hr may

have nothing to do with Dravet Syndrome. This work should be repeated with a hyperthermia treatment

that is relevant to the febrile seizures of Dravet Syndrome rather than heat stroke.2

We absolutely agree that hyperthermia to this degree is outside the physiological range to compare to

febrile seizures in human patients. This range was used based on the data from the extant literature

evaluating DS models.

However, outside of the issue of the degree of hyperthermia, we more broadly agree with the thoughtful

critiques of rev 1 and 2 that hyperthermia priming itself makes the data more difficult to interpret - is the

activation due to having gone through hyperthermia priming and a hyperthermic seizure? Or due to the

spontaneous seizure? Is there something different about these spontaneous seizures after hyperthermia

than about a true spontaneous seizure?

For those reasons we have re-done our experiments as described in the text - watching animals for a

spontaneous seizure without any exposure to the hyperthermia paradigm. We have largely similar findings

and I believe this experimental approach greatly strengthens our argument. We are very thankful to the

reviewers for this improvement. Please see this data in Figure 1.

All data from the hyperthermia priming have been removed.

2. In Figure 1C, the wild-type and DS Het values are labeled “Post-Seizure”. Did wild-type mice have seizures

during the treatment at 42.5 degrees C or in the 24 to 72 hr recording period afterwards?

The wildtype animals did not experience any seizures during the 42.5 degree C treatment, nor in the recording

afterwards. We apologize for the unclear labeling of this figure. The larger critique about the hyperthermic

seizure model is addressed above.

3. The statistical tests used to evaluate the significance of the data are not described. The data points in the

scatter plots presented in Figure 2C, Figure 3B, Figure 3C, or Figure 4J do not appear to be normally

distributed. If Student’s t-test or other tests designed for parametric statistics were used, the results of the

statistical analysis would not be valid for these apparently nonparametric distributions of data points. This

would cast doubt on most of the conclusions of this work. Please test these data sets for parametric distribution

of the data points, and if the distribution is nonparametric please use appropriate statistical test to determine

the significance of the differences observed.

We are very embarrassed and apologetic that this was not included in the methods as this is a major oversight

and make interpretation of our conclusions impossible, as you are state. There is now a “Experimental design

and statistical analysis” section that clearly notes - “A Gaussian distribution was not assumed; all comparisons

are nonparametric unpaired t-tests (Mann-Witney U test) used to determine statistical significance. Mean{plus minus}SEM

is provided throughout the text. Scatter plots are shown for all data with mean and SEM graphically depicted.”

4. Discussion. Decreased Excitability in BNST to PBN Projection Neurons. Lines 8-12. A simpler explanation

here is that these GABAergic projection neurons have reduced excitability due to loss of Nav1.1 channels, as

was shown previously for cerebellar Purkinje cells that are also GABAergic projection neurons (PMID:

17928448).

Thank you for this reference. It has been added as well as this potential explanation. We do feel that these

projection neurons are unlikely to be PV neurons or contain scn1a - see our RNAscope experiments in Figure

4-1.3

5. Discussion. Functional Implications. This section is long and speculative. It should be shortened greatly as

there is no supporting evidence for the points that are made. In addition, PBN connections to CV regulation

should be considered as a contributing factor in SUDEP (eg., PMID: 25477821).

Some of the more speculative points have been removed and the reference has been added, thank you.

REV.#2.

1. Word choice:

• Dravet syndrome should be described as a developmental and epileptic encephalopathy.

• “Mutation” should be replaced with “variant.”

• SCN1A encodes the sodium channel α subunit Nav1.1, not 1A.

• “Killed” should be replaced with “euthanized.”

• “Lower” RMP should be replaced with “more negative” or “hyperpolarized.”

This inconsistent and confusing language has been corrected.

2. The rationale for selection of the BNST over the CeA is not clear. Why choose this region over the CeA,

which has been linked to apnea upon seizure spread to that nucleus. Is there is evidence for similar effects in

the BNST, or whether stimulation or lesioning of the BNST, for example, leads to apnea or alterations in

breathing?

We absolutely agree that more thoroughly linking the BNST to peri-ictal apneas would be important and

powerful. There are preliminary experiments that address this question ongoing in our laboratory. However, we

believe even without this direct evidence of involvement of this region in ictal apneas there is rationale for

studying the BNST in a DS model. The BNST is intimately linked to both phasic fear behavior as well as

sustained anxiety behavior in rodents. The BNST has a role in social behavior, feeding behavior and dense

integration with the autonomic nervous system. DS has comorbid anxiety, mood disorders, social dysfunction,

dysautonomia and patient reported nutritional and feeding issues, so outside of any role in apnea, arousal and

SUDEP this is a region where neurophysiologic alterations could have impacts on disease - as summarized in

the discussion.

3. Description of the DS model: The authors should provide a more complete characterization of the DS mouse

phenotype, which is known to vary between laboratories. How often did the DS mice have spontaneous

seizures (the text only indicates that they were “infrequent”)? What were the frequency and age of onset of

sudden death?

3 cont -- Did the DS mice seize between the priming period and the monitoring period, and how often? Did the

primed mice experience lower-severity seizures prior to the tonic-clonic seizure, after which the animals were

euthanized for analysis? Were there DS mice that did not seize in response to the priming? Finally, in many

laboratories, DS mice die during the first tonic-clonic seizure. Thus, the feasibility of maintaining the animals for

1 hour following the tonic-clonic seizure requires additional explanation. How many animals died during the

tonic-clonic seizure and thus were not analyzed in the study? The study could be strengthened by the inclusion

of video/EEG data for at least some of the mice.

The DS model used here has been used in many other labs and well-characterized. We do agree that there is

often variability between laboratories and some characterization in our hands would go a long way to

strengthen our arguments. Please see Table 1, showing ages for those spontaneous seizures we captured, 4

none of which were associated with a death. There were also sudden deaths that did occur which were not

observed, and ages for these events are also now included. Our survival rates and data are similar to other

published reports using this model.

4. A critical question is whether spontaneous seizures and hyperthermia-induced seizures have the same

origin or activate the same brain circuits/pathways. If not, the changes observed here in the dBNST may be

applicable only to hyperthermia-induced seizures and may not be generally applicable to DS in which children

do not always present with febrile seizures. According to the c-fos data, which areas of the brain are primary

targets of the febrile seizures? Low-res images of c-fos staining in the brain slice would be helpful to

understand this question.

5. Why were the age ranges for priming different between genotypes?

6. Figure 1:

• The text states that spontaneous seizures were observed 24-72 h after priming, but the figure shows 24-48 h.

Which is correct?

• Fig. 1B is described as “Representative images of c-fos in dBNST and striatum in DS and WT mice 1 h post-seizure.” This sentence implies that WT mice seized, which is apparently not the case. More importantly, the

figure is incomplete. DS mice should be analyzed for c-fos expression in the absence of hyperthermic seizure

priming. Comparisons should be shown for each genotype in the primed and unprimed state, for animals that

were primed but analyzed prior to a tonic-clonic seizure, as well as for DS animals that had unprimed

spontaneous seizures vs. those that did not. It is described in the text that increased activation “appears to

result from seizures” - this statement would be more convincing with comparison to images of WT animals that

had also been primed with hyperthermia for the same length of time as DS to indicate whether priming leads to

dBNST changes unrelated to the seizure event itself.

These points largely discuss the pitfalls of the hyperthermia priming paradigm we initially used in order to

capture spontaneous seizures. As noted in response to reviewer one above, we agree with the thoughtful

critiques of rev 1 and 2 that hyperthermia priming makes the data more difficult to interpret. We have redone our experiments as described in the text (see Figure 1) - watching animals for a spontaneous

seizure without any exposure to the hyperthermia paradigm. Low-res images of c-fos staining in coronal

brain slices are now included, containing the BNST and dorsal striatum as well as hippocampus and

cortical regions showing expected c-fos activation.

7. The representative image shows more c-fos immunoreactivity in the (presumed) striatum of DS mice

compared to WT, although the signal intensity of these cells is not high and is unlikely to indicate strong

activation in this region post-seizure. However, please describe how images were thresholded, or how a

determination of a positive vs. negative cell was made in all images. Was this performed using the striatum

images for each genotype?

Additional detail is now provided in the text.

8. It is not clear to the reader that images were actually acquired from the striatum. There are no landmarks. A

low-res fluorescent image that includes a boxed-in region with higher magnification would be more convincing

than a cartoon. Alternatively, is there a specific antibody marker for this brain region?

Low-res images are now included, making the anatomy easier to determine.5

9. As described above, it is not clear what is meant by the baseline measurements in Fig. 1C.

This was referencing animals that had experienced handling but not hyperthermia protocol. These

experiments have been removed from the text as previously discussed.

10. In methods: What is meant by 95 mM ACSF? This is not a standard description. Also, why were

spontaneous and evoked sEPSCs recorded using different internal solutions?

Apologize for the typo - it was meant to read 95 mM CsF. This has been corrected and I agree that this

was a very unfortunate typo/autocorrect that resulted in a real inability to evaluate the electrophysiology.

11. Regarding the retrobead injection experiments:

• The header “dBNST neurons projecting to the PBN are a more excitable population,” needs come

clarification. More excitable than what?

• What neurons are represented by the non-fluorescent population data? The authors state that “all

physiologically distinct types of neurons ... were represented in these populations.” It is questionable whether a

sample size of n = 10 can accurately gauge the full set of neuronal types.

• The authors find that DS neurons projecting to the PBN are hypoexcitable, with a hyperpolarized RMP but no

other AP changes. Does this imply altered K+ currents?

-Header clarified - “dBNST neurons projecting to the PBN are a more excitable population than non-PBN

projecting neurons”.

-More detail has been provided about neuronal subtypes in the BNST and further experiments have been

performed to increase the n, with similar results. As noted now in the text, previous reports indicate that

neurons in the rat and mouse BNST can be categorized into at least three distinct neuronal subtypes based on

voltage responses to transient current steps (Egli and Winder, 2003; Hammack et al., 2007). All of these

physiologically distinct types of neurons were represented in the population of BNST neurons projecting to the

PBN, suggesting that changes in excitability in these projection neurons are not simply an enrichment for one

type. The plurality of these were Type I neurons (n=6/15 cells) but Type II (n=2 cells), Type III (n=4 cells) and

other (n=3 cells) are present. The fact that there is not a strict predominance of a single physiological type in

those neurons projecting to the PBN is not unexpected as neither neuronal morphology, expression of

neuropeptide markers, nor projection location has previously been shown to associate with a particular

physiological sub-type (Ch’ng et al., 2019; Rodríguez-Sierra et al., 2013; Silberman et al., 2013).

-Agreed, this possible interpretation has been added to the text.

12. The authors should explain/cite the rationale for recording AMPA-R and NMDA-R mediated currents at

different holding potentials. They should also provide reversal potentials for these measured currents.

This method is more clearly explained with references for reversal potential and further references using this

same method for determining AMPA and NMDA-R mediated currents using this same experimental setup and

internal solution in both the hippocampus and striatum.

13. The data in Fig. 3A and 3C seem to depend on outliers (especially panel B). Is this the case and do the

data remain significant if these few points are taken out of the analysis?6

We would first like to address and apologize for the lack of properly explained statistical methods, as noted

also by reviewer 1. There is now a “Experimental design and statistical analysis” section.

As for the data in Fig 3B - as this data does not follow a normal distribution outlier tests such as Dixon’s or

Grubb’s would not be appropriate to use to determine outliers (when using these tests, no points were found to

be outliers, but the data does not pass all tests for normality). In lieu of outlier testing, we used a cutoff of

values >95% of the CI to remove outliers, which includes the two highest data points in Fig 3B. After removing

these potential outliers, the comparison between WT and DS hets using a Mann-Whitney test produces a p

value of 0.059, which does change the interpretation. Similarly, removing the outlier in figure 3C by this

method also changes the p-value to 0.056.

Changes have been made to the figures and text, discussing the removal of outliers, and the revised p-values

now speaking towards a trend of an altered E/I ratio and a decrease in sIPSC frequency. We would like to note

that the K-S test in the cumulative probability plot remains significant with the removal of this data. We would

like to thank the reviewer for helping to strengthen and clarify conclusions by removing outlier data. Overall, the

lack of clearly significant findings is likely due to the heterogeneity of the neuronal population and we feel the

near significance and powerful trend to be important findings in their own right and does not diminish the

overall findings.

14. The authors could strengthen their work by directly assessing changes in postsynaptic receptor function or

expression through the application of receptor agonists (AMPA, NMDA or GABA). This experimental strategy

would bypass any presynaptic effects. Also, this experiment would allow pharmacological isolation of AMPAR- and NMDAR-mediated current components.

We fully agree that more experiments could be done to strengthen these conclusions. Micro/pico-spritzing of

receptor agonists in close proximity to the recorded neuron would not sufficiently exclude presynaptic

activation or extrasynaptic activation. We feel that an experimental technique that would fully and

unequivocally address this would require uncaging of glutamate using one or two photon systems, which we

feel are beyond the scope of this manuscript and not essential to the underlying conclusions.

15. Reduced sIPSC frequency indicates reduced release of GABA from the presynaptic terminals, suggesting

altered release probability. This result appears to be inconsistent with the paired-pulse recordings, which show

no significant changes. How do the authors explain these results?

This is correct, given the strong trend for a decrease in spontaneous IPSC frequency we would expect a trend

for an increased in paired-pulse ratio, reflecting decreased release probability. Paired-pulse measurements are

evoked by electrical stimulation, and recent reports suggest that evoked and spontaneous neurotransmission

processes are partially segregated at inhibitory synapses in the hippocampus (Horvath, P. M., Piazza, M. K.,

Monteggia, L. M., & Kavalali, E. T. (2020). Spontaneous and evoked neurotransmission are partially

segregated at inhibitory synapses. eLife, 9.) - thus there may be some inhibitory synapses only activated by

evoked neurotransmission, which may wash out the trend seen with the spontaneous IPSC data.

16. A major confound in the design of this study is stated by the authors: “The somewhat contradictory nature

of this is likely due to the heterogeneity of the BNST neurons and the inability to distinguish between

interneurons or projection neurons when doing blind-patching experiments-there may be a population within

the dBNST that has a more striking enhancement in intrinsic excitability if they were to be targeted directly.

Much of the dBNST neurons are GABAergic, with both interneurons and inhibitory projection neurons. In other 7

brain regions in DS models, inhibitory neurons, particularly PV interneurons, have greatly diminished

excitability and changes to AP kinetics. This was not seen overall in the BNST, likely due to the very small

population of PV interneurons.” I agree with the authors’ assessment. The inability to distinguish neuronal

identifies, combined with the small n numbers in the recording experiments, make it nearly impossible to

interpret the data. This study needs to be repeated using mice that are crossed to a fluorescent reporter for

PV+ interneurons, e.g. PV-td-Tomato. In addition, it would strengthen the study to measure changes in sodium

current density in acutely isolated PV+ dBNST neurons between genotypes. Finally, it would be helpful to stain

these regions for Nav1.1 in WT mice to understand the impact of Scn1a haloinsufficiency.

We appreciate the suggestion. There are very few, if any, PV interneurons in the dorsal or ventral BNST. In

agreement with prior literature (see the referenced Nguyen et al., 2016 using the PV-td-Tomato mouse) we

now present our own data evaluating the number of PV interneurons and presence of Scn1a in the BNST in

both WT and DS hets using RNAscope (see Figure 4-1), showing similar results to what has previously been

reported with the suggestion that there is very little, if any, PV neurons in the dBNST itself.

We agree studying more restricted neuronal populations is important, and we will continue down this avenue of

study in other neuronal subtypes but feel it is outside the scope of the current manuscript.

Minor comments:

1. As described in the methods, 4-5 male mice were approved to be co-housed in holding cages. Shouldn’t

male mice be housed separately?

2. A citation is needed at the end of p. 8 regarding the reversal potentials for the AMPAR-mediated and

NMDAR-mediated EPSCs, respectively.

3. The electrophysiology figures are very crowded and difficult to read.

4. The font in Fig. 5B and C is vanishingly small.

5. Were mice of both sexes used in the study?

-4-5 mice of the same sex were co-housed together, including males; this is consistent with how other groups

using the same mouse model house them and we likewise have no issues with aggression (Calhoun et al.

2017; Favero et al. 2018).

-Citations have been added, as noted above.

-Readability to figure 5 including font size has been addressed

-Yes, animals of both sexes were used. Again, we are very apologetic about the glaring omission of an

experimental and statistical design section in the methods, which has been remedied.

## References

[B1] Abdala AP, Toward MA, Dutschmann M, Bissonnette JM, Paton JFR (2016) Deficiency of GABAergic synaptic inhibition in the Kölliker-Fuse area underlies respiratory dysrhythmia in a mouse model of Rett syndrome. J Physiol 594:223–237. 10.1113/JP270966 26507912 PMC4704510

[B2] Amakhin DV, Malkin SL, Ergina JL, Kryukov KA, Veniaminova EA, Zubareva OE, Zaitsev AV (2017) Alterations in properties of glutamatergic transmission in the temporal cortex and hippocampus following pilocarpine-induced acute seizures in Wistar rats. Front Cell Neurosci 11:264. 10.3389/fncel.2017.00264 28912687 PMC5584016

[B3] Bernard C (2015) Spreading depression: epilepsy’s wave of death. Sci Transl Med 7:282fs14. 10.1126/scitranslmed.aaa9854 25855491

[B4] Bravo E, Marincovich A, Teran F, Crotts M, Lefkowitz RJ, Richerson G (2020) Postictal modulation of breathing by the central amygdala (CeA) can induce sudden unexpected death in epilepsy (SUDEP). FASEB J 34:1-1. 10.1096/fasebj.2020.34.s1.09947

[B5] Campos CA, Bowen AJ, Roman CW, Palmiter RD (2018) Encoding of danger by parabrachial CGRP neurons. Nature 555:617–622. 10.1038/nature25511 29562230 PMC6129987

[B6] Cassell MD, Freedman LJ, Shi C (1999) The intrinsic organization of the central extended amygdala. Ann N Y Acad Sci 877:217–241. 10.1111/j.1749-6632.1999.tb09270.x 10415652

[B7] Cheah CS, Frank HY, Westenbroek RE, Kalume FK, Oakley JC, Potter GB, Rubenstein JL, Catterall WA (2012) Specific deletion of NaV1. 1 sodium channels in inhibitory interneurons causes seizures and premature death in a mouse model of Dravet syndrome. Proc Natl Acad Sci U S A 109:14646–14651. 10.1073/pnas.1211591109 22908258 PMC3437823

[B8] Ch’ng SS, Fu J, Brown RM, Smith CM, Hossain MA, McDougall SJ, Lawrence AJ (2019) Characterization of the relaxin family peptide 3 receptor system in the mouse bed nucleus of the stria terminalis. J Comp Neurol 527:2615–2633. 10.1002/cne.24695 30947365

[B9] Davern PJ (2014) A role for the lateral parabrachial nucleus in cardiovascular function and fluid homeostasis. Front Physiol 5:436. 10.3389/fphys.2014.00436 25477821 PMC4235290

[B10] Derera ID, Delisle BP, Smith BN (2017) Functional neuroplasticity in the nucleus tractus solitarius and increased risk of sudden death in mice with acquired temporal lobe epilepsy. eNeuro 4:ENEURO.0319-17.2017. 10.1523/ENEURO.0319-17.2017 PMC5661358 29085908

[B11] Dlouhy BJ, Gehlbach BK, Kreple CJ, Kawasaki H, Oya H, Buzza C, Granner MA, Welsh MJ, Howard MA, Wemmie JA, Richerson GB (2015) Breathing inhibited when seizures spread to the amygdala and upon amygdala stimulation. J Neurosci 35:10281–10289. 10.1523/JNEUROSCI.0888-15.2015 26180203 PMC4502266

[B12] Dong H-W, Swanson LW (2004) Organization of axonal projections from the anterolateral area of the bed nuclei of the stria terminalis. J Comp Neurol 468:277–298. 10.1002/cne.10949 14648685

[B13] Dong H-W, Swanson LW (2006) Projections from bed nuclei of the stria terminalis, dorsomedial nucleus: implications for cerebral hemisphere integration of neuroendocrine, autonomic, and drinking responses. J Comp Neurol 494:75–107. 10.1002/cne.20790 16304681 PMC2707828

[B14] Dutschmann M, Herbert H (2006) The Kölliker-Fuse nucleus gates the postinspiratory phase of the respiratory cycle to control inspiratory off-switch and upper airway resistance in rat. Eur J Neurosci 24:1071–1084. 10.1111/j.1460-9568.2006.04981.x 16930433

[B15] Dutton SBB, Dutt K, Papale LA, Helmers S, Goldin AL, Escayg A (2017) Early-life febrile seizures worsen adult phenotypes in Scn1a mutants. Exp Neurol 293:159–171. 10.1016/j.expneurol.2017.03.026 28373025 PMC5538963

[B16] Egli RE, Winder DG (2003) Dorsal and ventral distribution of excitable and synaptic properties of neurons of the bed nucleus of the stria terminalis. J Neurophysiol 90:405–414. 10.1152/jn.00228.2003 12649311

[B17] Englot DJ, Morgan VL, Chang C (2020) Impaired vigilance networks in temporal lobe epilepsy: mechanisms and clinical implications. Epilepsia 61:189–202. 10.1111/epi.16423 31901182 PMC7033006

[B18] Favero M, Sotuyo NP, Lopez E, Kearney JA, Goldberg EM (2018) A transient developmental window of fast-spiking interneuron dysfunction in a mouse model of Dravet syndrome. J Neurosci 38:7912–7927. 10.1523/JNEUROSCI.0193-18.2018 30104343 PMC6125809

[B19] Fernandes HB, Catches JS, Petralia RS, Copits BA, Xu J, Russell TA, Swanson GT, Contractor A (2009) High-affinity kainate receptor subunits are necessary for ionotropic but not metabotropic signaling. Neuron 63:818–829. 10.1016/j.neuron.2009.08.010 19778510 PMC2756730

[B20] Gataullina S, Dulac O (2017) From genotype to phenotype in Dravet disease. Seizure 44:58–64. 10.1016/j.seizure.2016.10.014 27817982

[B21] Guyenet PG, Stornetta RL, Souza GMPR, Abbott SBG, Shi Y, Bayliss DA (2019) The retrotrapezoid nucleus: central chemoreceptor and regulator of breathing automaticity. Trends Neurosci 42:807–824. 10.1016/j.tins.2019.09.002 31635852 PMC6825900

[B22] Hammack SE, Mania I, Rainnie DG (2007) Differential expression of intrinsic membrane currents in defined cell types of the anterolateral bed nucleus of the stria terminalis. J Neurophysiol 98:638–656. 10.1152/jn.00382.2007 17537902

[B23] Hedrick TP, Nobis WP, Foote KM, Ishii T, Chetkovich DM, Swanson GT (2017) Excitatory synaptic input to hilar mossy cells under basal and hyperexcitable conditions. eNeuro 4:ENEURO.0364-17.2017. 10.1523/ENEURO.0364-17.2017 PMC5714709 29214210

[B24] Hsieh PF, Watanabe Y (2000) Time course of c-FOS expression in status epilepticus induced by amygdaloid stimulation. Neuroreport 11:571–574. 10.1097/00001756-200002280-00028 10718316

[B25] Huffman AM, Calhoun J, Kearney J (2018) Activity mapping of seizures in a mouse model of Dravet syndrome. FASEB J 32:556.4.

[B26] Isom LL (2014) “It was the interneuron with the parvalbumin in the hippocampus!” “No, it was the pyramidal cell with the glutamate in the cortex!” Searching for clues to the mechanism of Dravet syndrome–the plot thickens: Dravet syndrome–it’s not only interneurons. Epilepsy Curr 14:350–352. 10.5698/1535-7597-14.6.350 25678872 PMC4325595

[B27] Kalume F, Yu FH, Westenbroek RE, Scheuer T, Catterall WA (2007) Reduced sodium current in Purkinje neurons from Na_V_1.1 mutant mice: implications for ataxia in severe myoclonic epilepsy in infancy. J Neurosci 27:11065–11074. 10.1523/JNEUROSCI.2162-07.2007 17928448 PMC6672849

[B28] Kalume F, Westenbroek RE, Cheah CS, Yu FH, Oakley JC, Scheuer T, Catterall WA (2013) Sudden unexpected death in a mouse model of Dravet syndrome. J Clin Invest 123:1798–1808. 10.1172/JCI66220 23524966 PMC3613924

[B29] Kim S-Y, Adhikari A, Lee SY, Marshel JH, Kim CK, Mallory CS, Lo M, Pak S, Mattis J, Lim BK, Malenka RC, Warden MR, Neve R, Tye KM, Deisseroth K (2013) Diverging neural pathways assemble a behavioural state from separable features in anxiety. Nature 496:219–223. 10.1038/nature12018 23515158 PMC6690364

[B30] Kim Y, Bravo E, Thirnbeck CK, Smith-Mellecker LA, Kim SH, Gehlbach BK, Laux LC, Zhou X, Nordli DR Jr, Richerson GB (2018) Severe peri-ictal respiratory dysfunction is common in Dravet syndrome. J Clin Invest 128:1141–1153. 10.1172/JCI94999 29329111 PMC5824857

[B31] Knight LK, Depue BE (2019) New frontiers in anxiety research: the translational potential of the bed nucleus of the stria terminalis. Front Psychiatry 10:510. 10.3389/fpsyt.2019.00510 31379626 PMC6650589

[B32] Kuo F-S, Cleary CM, LoTurco JJ, Chen X, Mulkey DK (2019) Disordered breathing in a mouse model of Dravet syndrome. Elife 8:e43387. 10.7554/eLife.43387 31025941 PMC6506208

[B33] Lacuey N, Hampson JP, Harper RM, Miller JP, Lhatoo S (2019) Limbic and paralimbic structures driving ictal central apnea. Neurology 92:e655–e669. 10.1212/WNL.0000000000006920 30635481 PMC6382368

[B34] Lebow MA, Chen A (2016) Overshadowed by the amygdala: the bed nucleus of the stria terminalis emerges as key to psychiatric disorders. Mol Psychiatry 21:450–463. 10.1038/mp.2016.1 26878891 PMC4804181

[B35] Li C, Pleil KE, Stamatakis AM, Busan S, Vong L, Lowell BB, Stuber GD, Kash TL (2012) Presynaptic inhibition of gamma-aminobutyric acid release in the bed nucleus of the stria terminalis by kappa opioid receptor signaling. Biol Psychiatry 71:725–732. 10.1016/j.biopsych.2011.11.015 22225848 PMC3314138

[B36] Lopez-Santiago L, Isom LL (2019) Dravet syndrome: a developmental and epileptic encephalopathy. Epilepsy Curr 19:51–53. 10.1177/1535759718822038 30838929 PMC6610375

[B37] Luster BR, Cogan ES, Schmidt KT, Pati D, Pina MM, Dange K, McElligott ZA (2019) Inhibitory transmission in the bed nucleus of the stria terminalis in male and female mice following morphine withdrawal. Addict Biol 25:e12748. 10.1111/adb.12748 30963693 PMC6785353

[B38] Mazzone CM, Pati D, Michaelides M, DiBerto J, Fox JH, Tipton G, Anderson C, Duffy K, McKlveen JM, Hardaway JA, Magness ST, Falls WA, Hammack SE, McElligott ZA, Hurd YL, Kash TL (2018) Metabolic mapping of downstream network activity following CNO-induced activation of hM3Dq in BNST VGAT neurons. Mol Psychiatry 23:1. 10.1038/mp.2017.253 PMC5468515 27956747

[B39] Miller AR, Hawkins NA, McCollom CE, Kearney JA (2014) Mapping genetic modifiers of survival in a mouse model of Dravet syndrome. Genes Brain Behav 13:163–172. 10.1111/gbb.12099 24152123 PMC3930200

[B40] Misra C, Brickley SG, Farrant M, Cull-Candy SG (2000) Identification of subunits contributing to synaptic and extrasynaptic NMDA receptors in Golgi cells of the rat cerebellum. J Physiol 524:147–162. 10.1111/j.1469-7793.2000.00147.x 10747189 PMC2269854

[B41] Mistry AM, Thompson CH, Miller AR, Vanoye CG, George AL Jr, Kearney JA (2014) Strain- and age-dependent hippocampal neuron sodium currents correlate with epilepsy severity in Dravet syndrome mice. Neurobiol Dis 65:1–11. 10.1016/j.nbd.2014.01.006 24434335 PMC3968814

[B42] Navarrete-Opazo AA, Cook-Snyder DR, Miller JR, Callison JJ, McCarthy N, Palkovic B, Stuth EAE, Zuperku EJ, Stucke AG (2020) Endogenous glutamatergic inputs to the parabrachial nucleus/Kölliker-Fuse complex determine respiratory rate. Respir Physiol Neurobiol 277:103401. 10.1016/j.resp.2020.103401 32036030 PMC7309965

[B43] Nguyen AQ, Dela Cruz JAD, Sun Y, Holmes TC, Xu X (2016) Genetic cell targeting uncovers specific neuronal types and distinct subregions in the bed nucleus of the stria terminalis. J Comp Neurol 524:2379–2399. 10.1002/cne.23954 26718312 PMC5359980

[B44] Nobis WP, Schuele S, Templer JW, Zhou G, Lane G, Rosenow JM, Zelano C (2018) Amygdala-stimulation-induced apnea is attention and nasal-breathing dependent. Ann Neurol 83:460–471. 10.1002/ana.25178 29420859 PMC5867259

[B45] Nobis WP, González Otárula KA, Templer JW, Gerard EE, VanHaerents S, Lane G, Zhou G, Rosenow JM, Zelano C, Schuele S (2019) The effect of seizure spread to the amygdala on respiration and onset of ictal central apnea. J Neurosurg 132:1313–1323. 10.3171/2019.1.JNS183157 30952127 PMC8022327

[B46] Park K, Kanth K, Bajwa S, Girgis F, Shahlaie K, Seyal M (2020) Seizure-related apneas have an inconsistent linkage to amygdala seizure spread. Epilepsia 61:1253–1260. 10.1111/epi.16518 32391925

[B47] Paxinos G, Franklin KBJ (2019) Paxinos and Franklin’s the mouse brain in stereotaxic coordinates. London: Academic.

[B48] Perrotti LI, Hadeishi Y, Ulery PG, Barrot M, Monteggia L, Duman RS, Nestler EJ (2004) Induction of ΔFosB in reward-related brain structures after chronic stress. J Neurosci 24:10594–10602. 10.1523/JNEUROSCI.2542-04.2004 15564575 PMC6730117

[B49] Pollak Dorocic I, Fürth D, Xuan Y, Johansson Y, Pozzi L, Silberberg G, Carlén M, Meletis K (2014) A whole-brain atlas of inputs to serotonergic neurons of the dorsal and median raphe nuclei. Neuron 83:663–678. 10.1016/j.neuron.2014.07.002 25102561

[B50] Regehr WG (2012) Short-term presynaptic plasticity. Cold Spring Harb Perspect Biol 4:a005702. 10.1101/cshperspect.a005702 22751149 PMC3385958

[B51] Reichard RA, Subramanian S, Desta MT, Sura T, Becker ML, Ghobadi CW, Parsley KP, Zahm DS (2017) Abundant collateralization of temporal lobe projections to the accumbens, bed nucleus of stria terminalis, central amygdala and lateral septum. Brain Struct Funct 222:1971–1988. 10.1007/s00429-016-1321-y 27704219 PMC5378696

[B52] Rhone AE, Kovach CK, Harmata GI, Sullivan AW, Tranel D, Ciliberto MA, Howard MA, Richerson GB, Steinschneider M, Wemmie JA, Dlouhy BJ (2020) A human amygdala site that inhibits respiration and elicits apnea in pediatric epilepsy. JCI Insight 5:e134852. 10.1172/jci.insight.134852 PMC7213805 32163374

[B53] Rodríguez-Sierra OE, Turesson HK, Pare D (2013) Contrasting distribution of physiological cell types in different regions of the bed nucleus of the stria terminalis. J Neurophysiol 110:2037–2049. 10.1152/jn.00408.2013 23926040 PMC3841931

[B54] Silberman Y, Matthews RT, Winder DG (2013) A corticotropin releasing factor pathway for ethanol regulation of the ventral tegmental area in the bed nucleus of the stria terminalis. J Neurosci 33:950–960. 10.1523/JNEUROSCI.2949-12.2013 23325234 PMC3566560

[B55] Tai C, Abe Y, Westenbroek RE, Scheuer T, Catterall WA (2014) Impaired excitability of somatostatin- and parvalbumin-expressing cortical interneurons in a mouse model of Dravet syndrome. Proc Natl Acad Sci U S A 111:E3139–48. 10.1073/pnas.1411131111 25024183 PMC4121787

[B56] Tsai M-S, Lee M-L, Chang C-Y, Fan H-H, Yu I-S, Chen Y-T, You J-Y, Chen C-Y, Chang F-C, Hsiao JH, Khorkova O, Liou H-H, Yanagawa Y, Lee L-J, Lin S-W (2015) Functional and structural deficits of the dentate gyrus network coincide with emerging spontaneous seizures in an Scn1a mutant Dravet Syndrome model during development. Neurobiol Dis 77:35–48. 10.1016/j.nbd.2015.02.010 25725421

[B57] Villas N, Meskis MA, Goodliffe S (2017) Dravet syndrome: characteristics, comorbidities, and caregiver concerns. Epilepsy Behav 74:81–86. 10.1016/j.yebeh.2017.06.031 28732259

[B58] Wang L, Shen M, Jiang C, Ma L, Wang F (2016) Parvalbumin interneurons of central amygdala regulate the negative affective states and the expression of corticotrophin-releasing hormone during morphine withdrawal. Int J Neuropsychopharmacol 19:pyw060. 10.1093/ijnp/pyw060 27385383 PMC5137277

[B59] Wang Y, Kim J, Schmit MB, Cho TS, Fang C, Cai H (2019) A bed nucleus of stria terminalis microcircuit regulating inflammation-associated modulation of feeding. Nat Commun 10:2769. 10.1038/s41467-019-10715-x 31235690 PMC6591327

[B60] Wyeth MS, Pelkey KA, Petralia RS, Salter MW, McInnes RR, McBain CJ (2014) Neto auxiliary protein interactions regulate kainate and NMDA receptor subunit localization at mossy fiber-CA3 pyramidal cell synapses. J Neurosci 34:622–628. 10.1523/JNEUROSCI.3098-13.2014 24403160 PMC3870939

[B61] Yu FH, Mantegazza M, Westenbroek RE, Robbins CA, Kalume F, Burton KA, Spain WJ, McKnight GS, Scheuer T, Catterall WA (2006) Reduced sodium current in GABAergic interneurons in a mouse model of severe myoclonic epilepsy in infancy. Nat Neurosci 9:1142–1149. 10.1038/nn1754 16921370

[B62] Yuan Y, O’Malley HA, Smaldino MA, Bouza AA, Hull JM, Isom LL (2019) Delayed maturation of GABAergic signaling in the Scn1a and Scn1b mouse models of Dravet syndrome. Sci Rep 9:6210. 10.1038/s41598-019-42191-0 30996233 PMC6470170

