# Supplementary figures and images for: Enhanced Synaptic Transmission in the Extended Amygdala and Altered Excitability in an Extended Amygdala to Brainstem Circuit in a Dravet Syndrome Mouse Model
Source: eNeuro. 2021 Jun 16;8(3):ENEURO.0306-20.2021. doi: 10.1523/ENEURO.0306-20.2021 (PMC8213443; doi:10.1523/ENEURO.0306-20.2021)

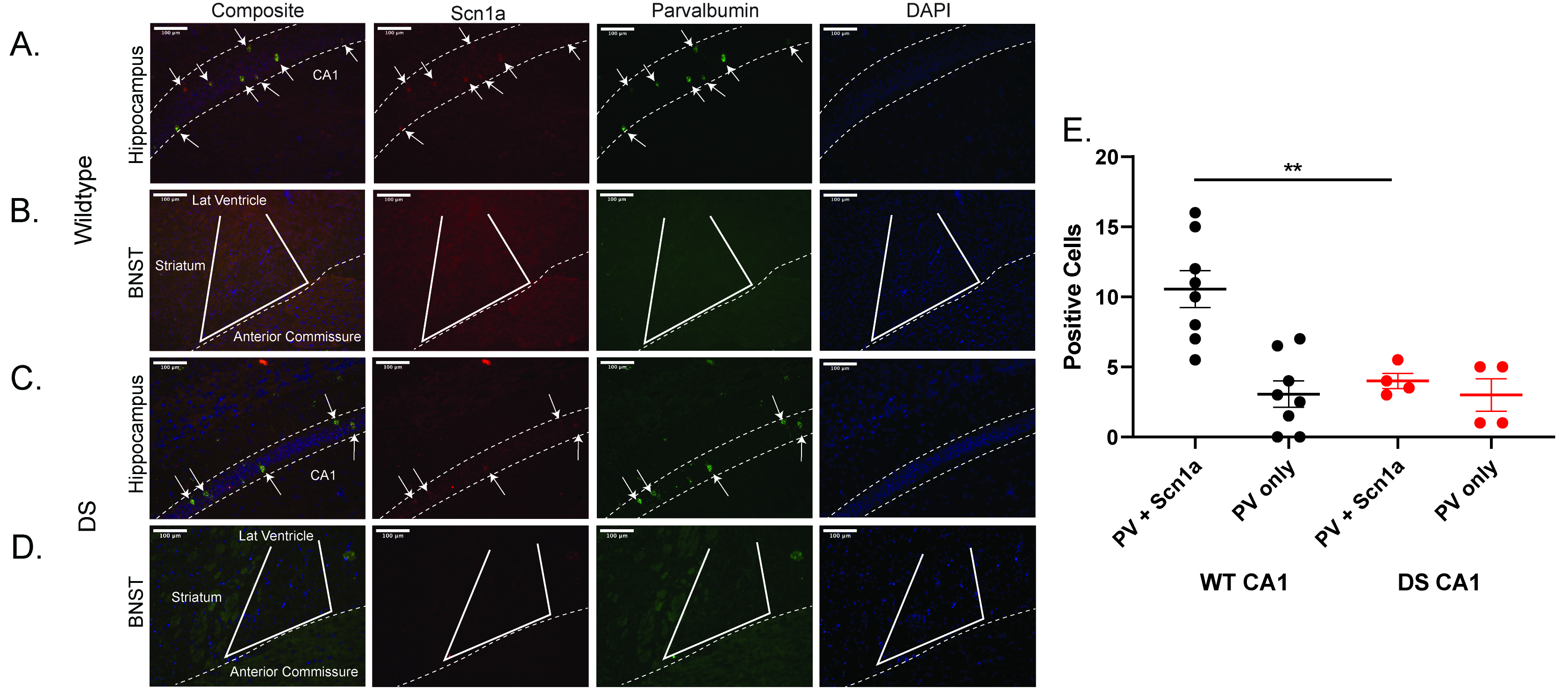

Supplement: Figure 5-1 — RNAScope of Scn1a and PV interneurons in hippocampus and BNST in DS and WT littermates. A, C, Scn1a colocalized with PV expression in the CA1 region of the hippocampus of both DS and WT mice with significantly lower Scn1a expression in DS mice. B, D, Scn1a and PV expression in the BNST is very sparse in both WT and DS mice. E, Quantification of Scn1a and PV expression in the hippocampus. Significant reduction in PV and Scn1a colocalized cells in CA1 hippocampal region of DS mice (WT, n = 8; DS, n = 4; p = 0.0069). Download Figure 5-1, TIF file. [file enu-eN-NWR-0306-20-s02.tif]
